# CXCR7: a β-arrestin-biased receptor that potentiates cell migration and recruits β-arrestin2 exclusively through Gβγ subunits and GRK2

**DOI:** 10.1186/s13578-020-00497-x

**Published:** 2020-11-23

**Authors:** Huong Thi Nguyen, Arfaxad Reyes-Alcaraz, Hyo Jeong Yong, Lan Phuong Nguyen, Hee-Kyung Park, Asuka Inoue, Cheol Soon Lee, Jae Young Seong, Jong-Ik Hwang

**Affiliations:** 1 Department of Biomedical Sciences, College of Medicine, Korea University, Seoul, Republic of Korea; 2 College of Pharmacy, University of Houston, Houston, TX USA; 3 Graduate School of Pharmaceutical Sciences, Tohoku University, Sendai, Japan

**Keywords:** SDF-1α, CXCR4, CXCR7, Biased GPCR, Structural complementation assay, Chemotaxis

## Abstract

**Background:**

Some chemokine receptors referred to as atypical chemokine receptors (ACKRs) are thought to non-signaling decoys because of their inability to activate typical G-protein signaling pathways. CXCR7, also known as ACKR3, binds to only two chemokines, SDF-1α and I-TAC, and recruits β-arrestins. SDF-1α also binds to its own conventional receptor, CXCR4, involving in homeostatic modulation such as development and immune surveillance as well as pathological conditions such as inflammation, ischemia, and cancers. Recently, CXCR7 is suggested as a key therapeutic target together with CXCR4 in such conditions. However, the molecular mechanisms underlying cellular responses and functional relation with CXCR7 and CXCR4 have not been elucidated, despite massive studies. Therefore, we aimed to reveal the molecular networks of CXCR7 and CXCR4 and compare their effects on cell migration.

**Methods:**

Base on structural complementation assay using NanoBiT technology, we characterized the distinct mechanisms underlying β-arrestin2 recruitment by both CXCR4 and CXCR7. Crosslinking and immunoprecipitation were conducted to analyze complex formation of the receptors. Gene deletion using CRISPR and reconstitution of the receptors were applied to analysis of ligand-dependent ERK phosphorylation and cell migration. All experiments were performed in triplicate and repeated more than three times. Unpaired Student’s *t-*tests or ANOVA using PRISM5 software were employed for statistical analyses.

**Results:**

Ligand binding to CXCR7 does not result in activation of typical signaling pathways via Gα subunits but activation of GRK2 via βγ subunits and receptor phosphorylation with subsequent β-arrestin2 recruitment. In contrast, CXCR4 induced Gα_i_ activation and recruited β-arrestin2 through C-terminal phosphorylation by both GRK2 and GRK5. SDF-1α-stimulated ERK phosphorylation was facilitated by CXCR4, but not CXCR7. Heterodimerization of CXCR4 and CXCR7 was not confirmed in this study, while homodimerization of them was verified by crosslinking experiment and NanoBiT assay. Regarding chemotaxis, SDF-1α-stimulated cell migration was mediated by both CXCR4 and CXCR7.

**Conclusion:**

This study demonstrates that SDF-1α-stimulated CXCR7 mediates β-arrestin2 recruitment via different molecular networking from that of CXCR4. CXCR7 may be neither a simple scavenger nor auxiliary receptor but plays an essential role in cell migration through cooperation with CXCR4.

## Introduction

Chemokine receptors, members of the G-protein-coupled receptor (GPCR) superfamily, induce directed cell migration toward their cognate ligands and therefore play important roles in inflammatory responses, homeostasis such as tissue maintenance, and development [1]. This subgroup of GPCRs consists of approximately 20 proteins, and more than 40 peptides, that have been identified by their chemokine ligand binding in humans [2, 3]. Most chemokine receptors interact with more than one chemokine, and in some cases, share ligands with other chemokine receptors. This interaction causes a conformational change, which stimulates cellular responses through canonical heterotrimeric G-protein activation [4]. However, other receptors, referred to as atypical chemokine receptors (ACKRs), cannot activate G-protein signaling, even though they bind many chemokines. This suggests that they may function as scavengers to modulate chemokine responses by unknown mechanisms [5]. Primary structure analysis has revealed that ACKRs share essential structural features for ligand binding with conventional chemokine receptors. However, there are some alterations in chemokine receptor-specific consensus sequences, including the DRYLAIV motif in the second intracellular loop, that are responsible for receptor/G-protein selectivity and for the efficiency of G-protein activation [6]. These structural differences between conventional chemokine receptors may provide unique biological roles under pathophysiological conditions, such as tissue shaping, inflammation, and cancer [6].

Unlike representative promiscuous ACKRs, such as DARC and D6, that interact with many chemokines, CXCR7 (also known as ACKR3) binds only two chemokines, SDF-1α and I-TAC, which are the ligands for CXCR4 and CXCR3, respectively [7]. Notably, SDF-1α is expressed in a variety of tissues and is involved in homeostatic modulations, such as immune surveillance and tissue development, including the establishment of hematopoietic cells in bone marrow, blood vessel formation, and nervous tissue development, as well as in pathological conditions, such as inflammation, ischemia, tumor angiogenesis, and autoimmune diseases [7, 8]. These SDF-1α-induced pathophysiological processes have been attributed to interactions with its conventional receptor, CXCR4. However, the recent finding that SDF-1α also interacts with CXCR7 suggests more sophisticated regulation of chemokine function according to the expression pattern of its receptors [9].

CXCR7 expression has been detected in a variety of tissues by northern blot, RT-PCR, western blot, and immunohistochemistry analyses [10–13]. Notably, different immune-cell lines express CXCR7 at different levels, suggesting that it may be involved in immune-cell differentiation [14–17]. The promoter region of the *CXCR7* gene contains binding elements for transcription factors NF-κB and HIF-1α, which are also found in the *SDF-1*α and *CXCR4* genes, suggesting that these factors are necessary for optimal SDF-1α expression [18]. In contrast, the tumor suppressor Hypermethylated in Cancer 1 (HIC1) represses CXCR7 expression [19]. These transcriptional regulators may explain the increase in CXCR7 expression in many cancers, including breast, lung, cervical, myeloid, glial, and prostate [20–25]. Similar to CXCR4, the expression of CXCR7 would give cancer cells a metastasis advantage, by moving cells toward an SDF-1α gradient. CXCR7 expression is also upregulated in other pathological conditions such as inflammation, infection, and ischemia, suggesting that its expression is likely regulated by exogenous cues. For this reason, CXCR7 has been proposed as a potential prognostic marker for some pathological conditions [26, 27].

After CXCR7 was identified as another SDF-1α binding protein [9], CXCR7 functional studies have been the subject of intensive research. Notably, the high perinatal death rate in *CXCR7*-deficient mice (> 95%) was reported to be mainly due to cardiovascular defects and also correlated with CXCR7 expression during embryonic vascular development [13]. There is also increasing evidence that CXCR7 is an additional biological process modulator for angiogenesis and immune responses, even though CXCR4 has long been considered to be the main mediator of SDF-1α-dependent cell differentiation [28–31].

As an above-mentioned ACKR, the scavenging activity of CXCR7 for SDF-1α has been emphasized because it contains the DRYLSIT motif instead of the consensus DRYLAIV motif that is thought to be responsible for chemokine receptor-mediated Gα_i_ activation [6, 12]. However, this CXCR7 motif is also found in other GPCRs (e.g., adrenergic receptors, acetylcholine receptors, and serotonin receptors), suggesting that the motif itself does not explain impaired Gα_i_ activation [32]. Considerable effort has been made to establish the mechanisms underlying CXCR7 biology, and many reports have documented its biochemical properties. For example, CXCR7 has been reported to have a higher affinity for SDF-1α compared with CXCR4, and it easily internalizes and removes the chemokine from the extracellular milieu [9, 33]. Studies using receptor-specific antagonists, and receptor-overexpressing cells revealed that CXCR7 may not be implicated in SDF-1α-induced cellular responses, such as calcium mobilization and phosphorylation of both AKT and ERK1/2 [33]. However, in some cellular systems, CXCR7 has been proposed to mediate SDF-1α-stimulated ERK1/2 and AKT phosphorylation as well as activation of some types of protein kinase C. Notably, ERK1/2 phosphorylation was sensitive to pertussis toxin pretreatment, indicating that Gα_i_ may somehow be involved [34]. CXCR4 and CXCR7 have even been reported to form complexes with each other using a BRET-based titration assay, suggesting that CXCR7 may modulate CXCR4-mediated cellular responses [35], but another study showed that they were not co-internalized by the ligand, so heterodimer formation did not occur [14]. These conflicting data are likely due to the overexpression of these receptors, producing many non-specific interactions between the two receptors.

Despite these possible problems, CXCR7, similar to CXCR4, is known to interact with β-arrestin2 in a SDF-1α-dependent manner, indicating that CXCR7 is responsible for SDF-1α internalization and may induce β-arrestin2-mediated signaling events [36]. However, mediators for this receptor-β-arrestin2 interaction have not been elucidated yet. For these reasons, an integrative analysis of CXCR7-mediated signaling may provide clear answers to illustrate the biological properties of CXCR7 and make a case for this receptor as a therapeutic target.

In the present study, we characterized the distinct mechanisms underlying β-arrestin2 recruitment by both CXCR4 and CXCR7 through molecular-interaction analyses using a structural complementation assay. These biochemical studies revealed that SDF-1α-stimulated G-protein activation and related signaling events were mediated by CXCR4, but not by CXCR7. These receptors seemed to form homodimers, but not heterodimers with other receptors in the plasma membrane, which facilitated receptor-mediated signaling. Given the role of chemokine receptors in cell migration, it is likely that these receptors complement each other. This type of relationship was verified using cells either lacking or overexpressing these receptors in a chemotaxis assay. Overall, the results suggested that CXCR7 is not a simple auxiliary receptor but plays an essential role in cell migration through cooperation with CXCR4.

## Results

### CXCR7 recruits β-arrestin2 with higher efficacy in a ligand-dependent manner, compared with CXCR4 and CXCR3

**Fig. 1:**
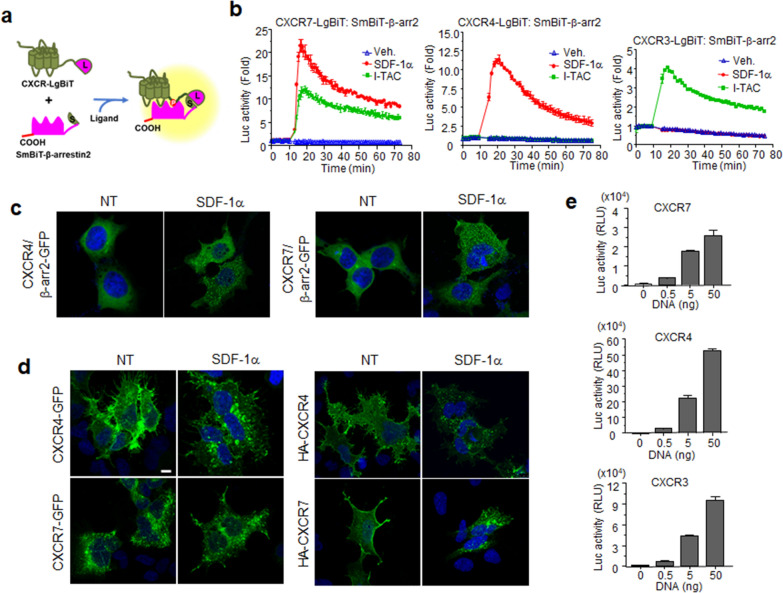
SDF-1α induced β-arrestin2 recruitment to CXCR4 and CXCR7. **a** Schematic representation of the structural complementation assays for the interaction of β-arrestin2 with chemokine receptors **b** HEK293 cells, transiently transfected with constructs for receptor-LgBiT and SmBiT-β-arrestin2, were treated with the indicated chemokines (I-TAC/SDF-1α) or not (Veh.), and then assessed for real-time luminescence. **c** CXCR4 and CXCR7 mediate intracellular clustering of β-arrestin2 by SDF-1α. Cells transfected with β-arrestin2-GFP and chemokine receptor constructs were treated with SDF-1α for 30 min. GFP fluorescence was then assessed using confocal microscopy. **d** Translocation of CXCR4 and CXCR7 to the cytosol by SDF-1α. Cells expressing CXCR4-GFP or CXCR7-GFP were treated with SDF-1α and fixed. Confocal images show the subcellular localization of GFP signals (left panels). Cells expressing HA-tagged receptors were labeled with anti-HA antibodies and treated with SDF-1α for 30 min (NT: no treatment). Fixed cells were permeabilized and stained with FITC-conjugated goat anti-mouse IgG. The images show the subcellular localization of the HA-tagged receptors (right panel). **e** Cell surface expression of the receptors. Transfected cells with constructs for HiBiT-tagged receptors were incubated with extracellular HiBiT detection reagent, and luminescence was measured. The results are an average of three independent experiments. Values are presented as the mean ± SD

CXCR7 shares ligands with both CXCR4 and CXCR3 [37], but its receptor-mediated signaling pathways are not completely understood. One of the clear responses elicited by CXCR7 is ligand-dependent β-arrestin2 recruitment. To explore signaling by this chemokine receptor, β-arrestin2 recruitment assays were developed on the basis of structural complementation using NanoBiT technology. This technology uses two separate fragments of Nano Luciferase (Nluc), a small protein that catalyzes a bright luminescent reaction. Binding of the small fragment (SmBiT) with the large fragment (LgBiT) of Nano Luciferase produces a bright luminescent signal, but these fragments normally have very low affinities for each other, so their close proximity, driven by the interactions of fusion partners, can be used to study protein–protein interactions [38]. If these interactions are reversible, then both the associations and dissociations of the targeted proteins can be monitored. There is no lag time for luminescence lost, so detection is immediate, and accurate temporal dynamics of the protein interactions can be determined [39]. To monitor β-arrestin2 recruitment at chemokine receptors, four different plasmid combinations were screened for β-arrestin2 fused with NanoBiT fragments (LgBiT or SmBiT at either the N-terminus or C-terminus) and two types (LgBiT or SmBiT) of the C-terminal-fused chemokine receptors (CXCR3, 4, and 7) (Fig. 1a). The plasmid combination that showed the best response was selected for further studies. Interactions between β-arrestin2 and the CXCRs were immediately observed after ligand stimulations, reaching maximal luminescence 5 min after ligand addition. All combinations of CXCR7 and β-arrestin2 showed an increase in luminescence by SDF-1α and I-TAC with different efficacies (Additional File 1: Fig. S1). CXCR4 combinations showed a wide variety of efficacies by SDF-1α treatment (Additional File 1: Fig. S2). Notably, CXCR4-SmBiT and LgBiT-β-arrestin2 showed no increase in luminescence signal. For CXCR3, the response patterns from I-TAC were very similar among all combinations (Additional File 1: Fig. S2). Neither CXCR3 nor CXCR4 showed any responses to SDF-1α or I-TAC respectively in all plasmid combinations, supporting previous reports that SDF-1α is not a ligand for CXCR3 and I-TAC is not a ligand for CXCR4 (Figs. 1b and Additional File 1: Fig. S2). CXCR7-mediated responses to SDF-1α and to I-TAC were much higher than CXCR3 and CXCR4 responses. SDF-1α induced higher luminescence signals than I-TAC in cells expressing CXCR7, indicating that SDF-1α induces receptor conformational changes favorable to β-arrestin2 binding in comparison to I-TAC (Fig. 1b).

β-Arrestin2 recruitment to the chemokine receptors precedes their internalization from the plasma membrane [40]. This internalization of CXCRs was monitored using β-arrestin2-GFP fusion proteins. As shown in Fig. 1c, β-arrestin2-GFP was localized to the cytosol in the absence of ligand. Upon SDF-1α treatment of cells expressing CXCR4 or CXCR7, GFP signals were clustered in the cytosol as aggregates, indicating that β-arrestin2 was translocated to specific regions of the cytosol, perhaps as early endosomes. CXCR4-GFP was mainly localized to the plasma membrane in the absence of ligand, and translocated to the cytosol after ligand treatment, whereas most CXCR7-GFP was detected in the cytosol regardless of ligand treatment, indicating cytosol retention that made assessment of receptor spatial locations difficult (Fig. 1d left panel). To examine receptor-only behavior in the plasma membrane, as described previously, cells expressing HA-tagged receptors were incubated with anti-HA antibodies prior to SDF-1α treatment [41]. We observed changes in the localization of CXCR4 from the plasma membrane to the cytosol after ligand treatment, and CXCR7 expression in the plasma membrane was weak but detectable, and its trafficking could be monitored after ligand treatment (Fig. 1c, right panel). Membrane expressions of chemokine receptors were further determined by HiBiT assay. Luminescence signals in cells expressing SmBiT-receptors became stronger depending on the amount of transfected plasmid. Luminescence in cells expressing CXCR7 was approximately tenfold or threefold lower than luminescence in CXCR4- or CXCR3-expressing cells, respectively (Fig. 1e). According to these data, CXCR7 seems to interact strongly with β-arrestin2 compared with CXCR4, even with poor plasma membrane localization. The strong affinity of CXCR7 toward β-arrestin2 may be due to a higher binding affinity to the ligand compared with CXCR4 or CXCR3, as described previously [9, 37], or the ligand-bound receptor may undergo conformational changes that are more suitable for β-arrestin2 interaction.

SDF-1α-dependent internalization of CXCR4 and CXCR7 was confirmed using two structural complementation assays based on NanoBiT technology. For the first assay, constructs receptor-LgBiT and SmBiT-FYVE domain were used, where the FYVE domain was used as an early endosome marker. The luminescence signal was significantly increased few minutes after ligand treatment, indicating that the receptor was internalized via early endosomes (Additional File 1: Fig. S3a). The second structural complementation assay was based on the plasma membrane marker CAAX. When a receptor is expressed in the plasma membrane, its close proximity to CAAX produces high luminescence. However, when a receptor is activated by a ligand, internalization occurs and the receptor is no longer in close proximity to CAAX, resulting in decreased luminescence. The luminescence signal of CXCR4-SmBiT and LgBiT-CAAX was decreased by SDF-1α (Additional File 1: Fig. S3b, left). The luminescence signal of CXCR7-SmBiT and LgBiT-CAAX was also decreased by SDF-1α, but recovered slightly after 30 min (Additional File 1: Fig. S3B, right), implying a dynamic localization of CXCR7 such as possibly recycling or translocation of cytosolic CXCR7. The fast recovery of CXCR7 to the membrane may have been due to dominant receptor localization to the cytosol as shown in Fig. 1d.

### CXCR4 and CXCR7 compete each other for SDF-1α

**Fig. 2:**
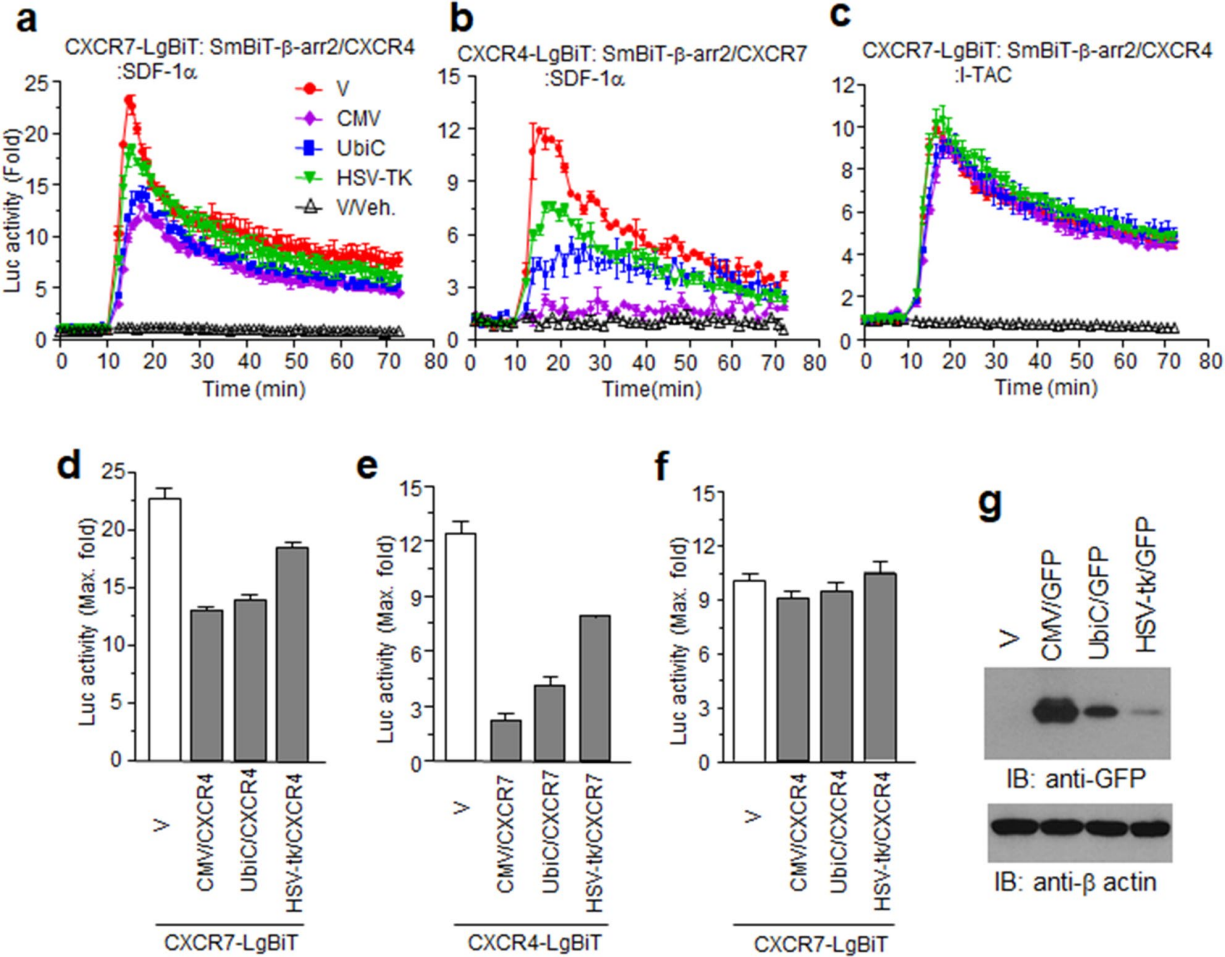
CXCR7 and CXCR4 compete with each other for SDF-1α ligand specificity. **a**–**c** NanoBiT receptor/β-arrestin2 constructs were co-expressed with other receptors governed by different promoters as described for each graph. **d**–**f** Graphs of maximum luminescence activities in each experiment from (**a**–**c)**. **g** Transcriptional activities of each promoter. HEK293 cells expressing GFP governed by the CMV (CMV/GFP), Ubiquitin C (UbiC/GFP), or herpes simplex virus-thymidine kinase (HSV-tk/GFP) promoter were lysed and used for western blotting with anti-GFP antibodies. Results are the average of three independent experiments. Values are presented as the mean ± SD

Like other ACKRs, CXCR7 has been suggested as a decoy, or scavenger, for chemokines. To investigate chemokine affinities and specificities, we constructed intact forms of CXCR4 and CXCR7 in plasmids with different promoters, and applied NanoBiT assays. The SDF-1α-stimulated luminescence signals for CXCR7-LgBiT and SmBiT-β-arrestin2 decreased depending on CXCR4 expression. However, these signals were sustained at approximately half-maximum even in the presence of high CXCR4 expression (Fig. 2a, d). In contrast, CXCR4-LgBiT and SmBiT-β-arrestin2 signals were remarkably decreased by the overexpression of CXCR7 (Fig. 2b, e), suggesting a stronger affinity between CXCR7 and SDF-1α compared with CXCR4. I-TAC-stimulated luminescence for CXCR7-LgBiT and SmBiT-β-arrestin2 was not affected by CXCR4 (Fig. 2c, f), indicating that chemokine binding to its cognate receptor is sufficient to distinguish it from other chemokine receptors. The different promoters induced different receptor-expression efficacies, as determined by GFP expression (Fig. 2g).

### CXCR7 is likely to form homodimers, but not heterodimers, with CXCR4

**Fig. 3:**
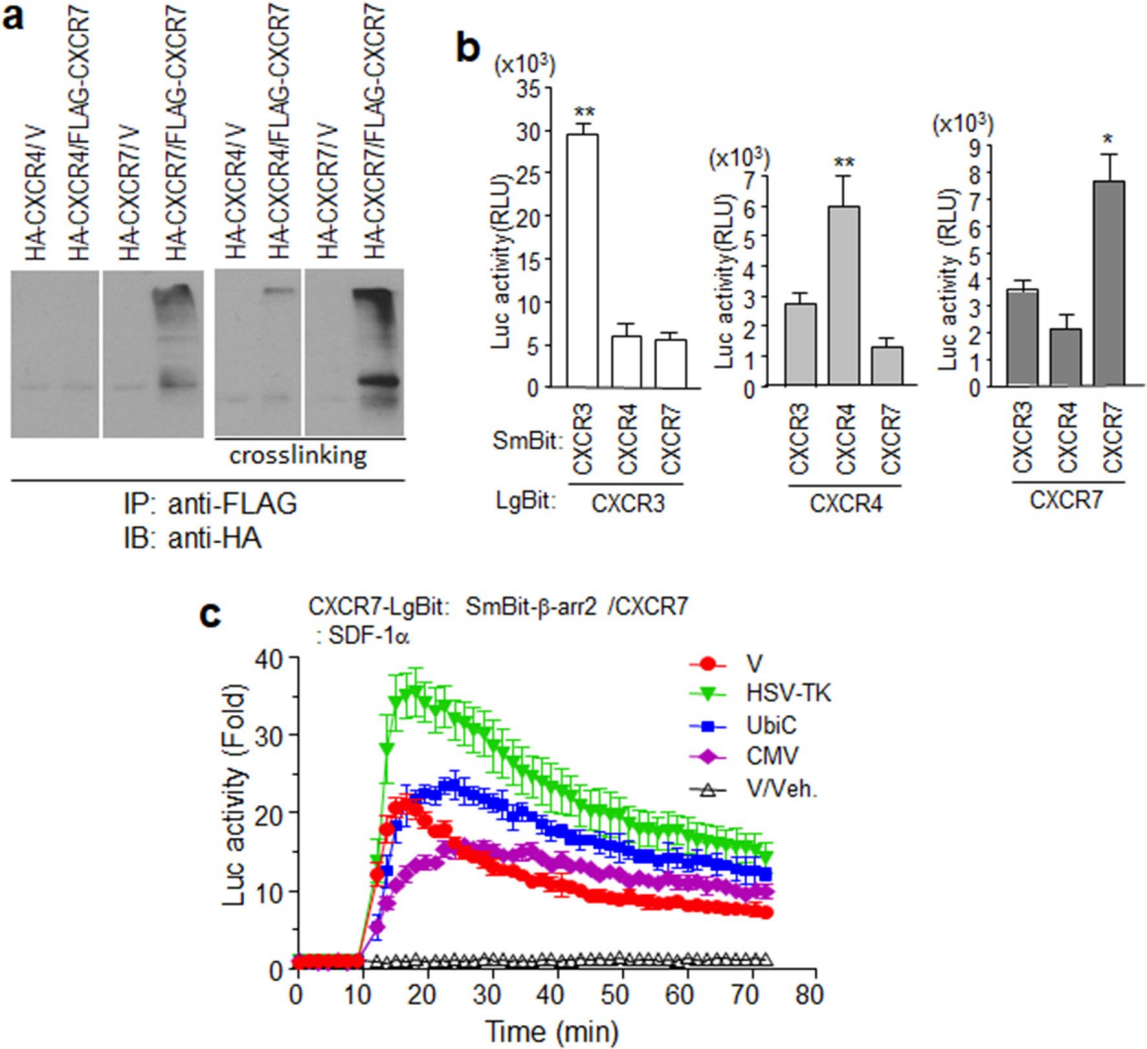
Homodimerization of CXCR7 on the membrane surface. **a** HEK293 cells expressing epitope-tagged forms of CXCR4 or CXCR7 were used for immunoprecipitation with anti-FLAG agarose and subsequent western blot analysis with anti-HA antibodies. Cross-linking experiments were performed as described in Materials and Methods. **b** Luminescence produced by receptor dimerization. NanoBiT constructs for each receptor were expressed in HEK293 cells. **c** NanoBiT constructs of CXCR7 and β-arrestin2 were co-expressed with different promoter constructs of CXCR7 (HSV-TK, UbiC, CMV) or empty vector (V) in HEK293 cells. Cells were treated with SDF-1α or not (V/Veh), and then luminescence was measured. Results are the average of three independent experiments. Values are presented as the mean ± SD. *p < 0.05, **p < 0.001

Previous studies have reported the possibility of heterodimerization between CXCR7 and CXCR4 [42, 43]. Therefore, this possible dimerization was expected to affect I-TAC-mediated CXCR7 signaling. The luminescence signals of CXCR7-LgBiT stimulated with both SDF-1α and I-TAC should have been changed by CXCR4 overexpression, but CXCR4 overexpression had no effect on I-TAC-stimulated CXCR7 β-arrestin2 recruitment (Fig. 2c). To determine heterodimerization between CXCR4 and CXCR7, cells expressing the receptors tagged with different epitopes for co-immunoprecipitation were used with anti-FLAG antibodies. Figure 3a shows that HA-CXCR7, but not HA-CXCR4, was co-precipitated with FLAG-CXCR7. To further confirm this, cells were incubated with a cross-linker for immunoprecipitation using RIPA buffer (for increased stringency to avoid non-specific interactions). More HA-CXCR7 was co-precipitated with the cross-linker than without it. A very weak HA-CXCR4 signal was detected at the starting line of the gel region. This might have been due to weak interactions between CXCR4 and CXCR7, or to artificial binding due to overexpression. A structural complementation assay, based on NanoBiT technology, was used to examine real-time membrane protein interactions in a living system. Both SmBiT and LgBiT forms of the receptors were co-expressed in the cell, and luminescent signals were measured at a single time point. Interestingly, combinations of the same receptor produced high luminescence, but combinations of different receptors produced low luminescence signals. These observations suggest that these receptors may be expressed in the plasma membrane as homodimers, rather than as heterodimers with other receptors (Fig. 3b).

Cells expressing both CXCR7-LgBiT and SmBiT-β-arrestin2, but with different promoter-driven intact CXCR7 expression, were treated with SDF-1α. In the presence of the intact CXCR7 under the HSV-TK promoter, SDF-1α-stimulated cells produced higher luminescent signals in comparison to those in the absence of intact CXCR7. In contrast, overexpressing intact CXCR7 with the CMV promoter decreased the luminescence (Fig. 3c). This suggests that since CXCR7s easily form homodimer in the plasma membrane (Fig. 3a, b), HSV-TK-driven intact CXCR7 may bind CXCR7-LgBiT to form dimer, which brings about an increase in the absolute number of CXCR7 dimer (CXCR7/CXCR7-LgBiT or CXCR7-LgBiT/CXCR7-LgBiT) being able to interact with SmBiT-β-arrestin2. However, overexpressed intact CXCR7 driven by CMV is dominant over CXCR7-LgBiT on SDF-1α binding.

### Gα subunits are dispensable for ligand-stimulated β-arrestin2 recruitment at CXCR7

**Fig. 4:**
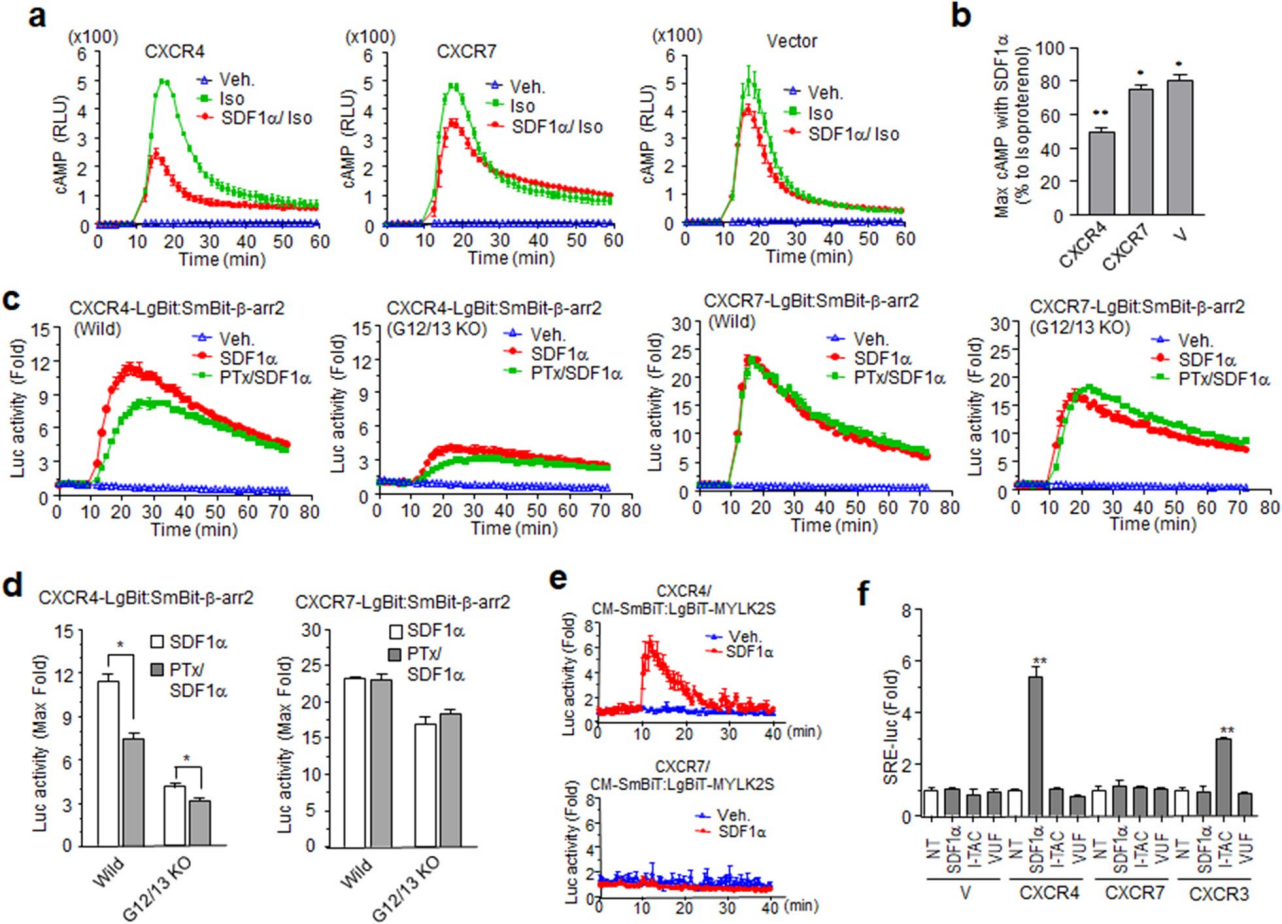
Characterization of receptor-mediated chemokine signaling events. **a** cAMP assay. HEK293 cells transfected with receptor gene plasmids or an empty vector (V) and pGlo22F plasmid containing a cAMP detector gene were incubated with Glosensor cAMP reagent. Cells were treated with SDF-1α for 10 min prior to isoproterenol (Iso) or no treatment (Veh.). Real-time intracellular cAMP production was measured using luminescence. **b** Each bar in the graph indicates the percent change between the SDF-1α-pre-treated group versus the maximal value of isoproterenol alone. **c** Effect of G-proteins on β-arrestin2 recruitment to the receptors. HEK293 cells (Wild) and Gα_12/13_-deficient HEK293 cells (G12/13 KO) were transiently transfected with the NanoBiT receptor constructs and β-arrestin2. After overnight incubation with and without pertussis toxin (PTx), cells were treated with SDF-1α or not (Veh.) and the luminescence determined. **d** Graphs of maximum luminescence in each experiment from **c**. **e** Receptor-mediated SRE-luc reporter gene expression by chemokines. HEK293 cells were transfected with each receptor constructs (CXCR4, CXCR7, CXCR3) or empty plasmid (V) and SRE-luc plasmids, and ligand-stimulated luminescence was measured (VUF11207 is a CXCR7 agonist, NT = no treatment). Results are the average of three independent experiments. Values are presented as the mean ± SD. *p < 0.05, **p < 0.001

Chemokine receptors stimulate Gα_i/o_ and probably Gα_12/13_ family members to induce cellular responses [44, 45]. To understand the functional mechanisms of CXCR7 in terms of cellular responses, we investigated early signaling events mediated by heterotrimeric G-proteins. Gα_i/o_, activated by GPCRs, inhibits Gα_s_-activated adenylyl cyclase. Cyclic AMP (cAMP) production was measured by real-time luminescence in the cells transfected with the receptor and with the Glosensor-22F plasmid. HEK293 cells endogenously express β-adrenergic receptors that activate the Gα_s_ pathway [46]. Isoproterenol-induced cAMP generation was remarkably decreased by SDF-1α pretreatment in cells expressing CXCR4, but only a slight decrease in cAMP levels was seen in cells expressing CXCR7 (Fig. 4a). Maximum isoproterenol-induced cAMP levels decreased by approximately 50% by SDF-1α in the presence of CXCR4, but by less than 20% in parent cells and CXCR7-expressing cells (Fig. 4b). This result raised the possibility that CXCR7 did not activate the Gα_i/o_ family.

To examine the effect of G-proteins on β-arrestin2 recruitment to the receptors, cells expressing NanoBiT constructs were pre-treated with pertussis toxin and used for NanoBiT assays with SDF-1α. The luminescence signals resulting from interactions between CXCR4 and β-arresin2 were significantly decreased by pertussis toxin, whereas interactions between CXCR7 and β-arrestin2 were not affected. When the same experiments were performed in Gα_12/13_-knockout cells, SDF-1α-stimulated luminescence of CXCR4 towards β-arrestin2 recruitment decreased prominently compared to wild-type cells. Signal reduction by pertussis toxin was also observed, even in these knockout cells. These results suggested that SDF-1α-stimulated β-arrestin2 recruitment to CXCR4 depends on both Gα_i/o_ and Gα_12/13_. The luminescence signals from interactions between CXCR7 and β-arrestin2 decreased slightly in the absence of Gα_12/13_ but were not affected by pertussis toxin. However, it is still unknown whether this change was due to the absence of Gα_12/13_ or the characteristics of cell cloning. Given the effect of Gα_12/13_ deficiency on interactions between CXCR4 and β-arrestin2, we suggest that both Gα families are dispensable for ligand-dependent β-arrestin2 recruitment towards CXCR7 (Fig. 4c, d).

Activation of chemokine receptors is also determined by intracellular calcium increase. Since the calcium mobilization through PLC-β interaction to Gβγ released from Gα_i/o_ is relatively weak, the chimeric G protein Gα_qi_ is feasible to detect Gα_i/o_ activation in calcium assay. NanoBiT-based calcium assay which is newly developed by us revealed that CXCR4 but not CXCR7 mediated SDF-1α-stimulated calcium increase in the cells expressing Gα_qi_ (Fig. 4e). Reporter gene assay is another powerful tool for detecting GPCR activation. Notably, SRE-driven luciferase expression by chemokine receptor activation can be examined in the presence of chimeric G-protein Gα_qi_ [47]. Increased luciferase activities were observed after SDF-1α or I-TAC treatment of cells expressing CXCR4 and CXCR3, respectively. However, neither the chemokines nor the specific agonist VUF11207 enhanced luciferase activity in cells expressing CXCR7, confirming that the Gα protein subunit is not involved in CXCR7-mediated cellular responses (Fig. 4f).

### SDF-1α-stimulated ERK1/2 phosphorylation is mediated by CXCR4 but not CXCR7

**Fig. 5:**
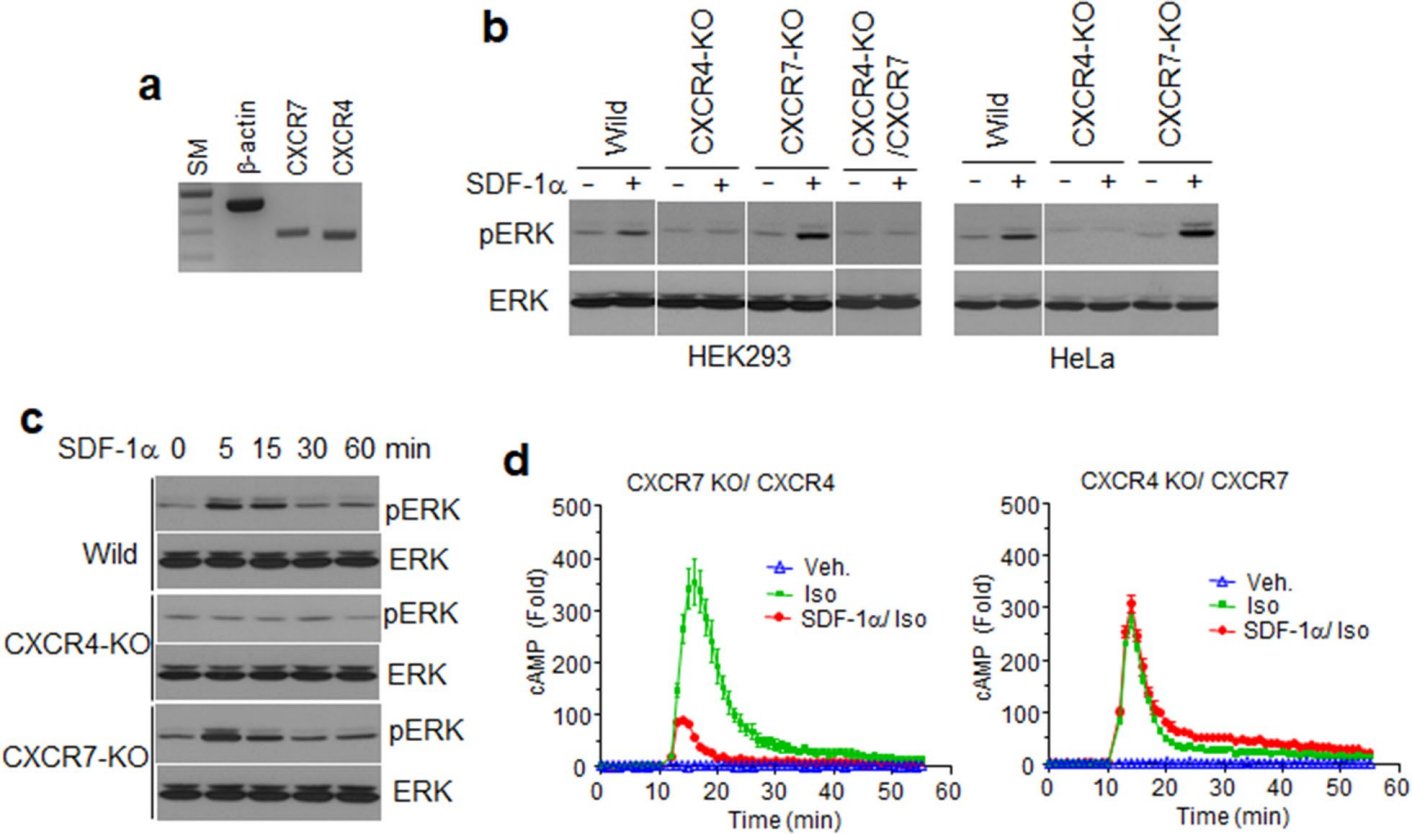
SDF-1α-stimulated ERK1/2 phosphorylation was mediated by endogenous CXCR4. **a** RT-PCR. RNA isolated from HEK293 cells was subjected to RT-PCR using gene-specific primer sets. The PCR products were separated using 2% agarose gels (SM: 1 kb ladder size marker). **b** HEK293 cells and HeLa cells lacking *CXCR4* (CXCR4-KO) or *CXCR7* genes (CXCR7-KO) were established by CRISPR/Cas9 gene-deletion methods. CXCR4-deficient HEK293 cells were transfected with CXCR7 plasmids (CXCR4-KO/CXCR7). Cells were treated with SDF-1α for 10 min and harvested. Cell lysates were used for western blot analysis with anti-pERK or ERK antibodies. **c** Time-dependent ERK phosphorylation by SDF-1α in wild-type HEK293 cells (wild), CXCR4- or CXCR7-deficient HEK293 cells (CXCR4-KO or CXCR7-KO). **d** The efficiency of SDF-1α inhibition on isoproterenol-stimulated cAMP generation in CXCR4- or CXCR7-deficient HEK293 cells. The cells were transfected with receptor gene plasmids and pGlo22F containing a cAMP detector gene plasmid. Cells were treated with SDF-1α for 10 min prior to isoproterenol (Iso) or no treatment (Veh.). Real-time intracellular cAMP production was measured as luminescence. Results are the average of three independent experiments.

Established cell lines express a wide variety of transmembrane membrane receptors to survive and to respond to extracellular stimuli. Detection of CXCR4 has previously been reported in HEK293 cells at the mRNA level [48]. As endogenously expressed GPCRs are rarely detected using antibodies (due to the amount of protein and antibody quality), we investigated CXCR4 and CXCR7 by RT-PCR. A specific CXCR4 PCR product was detected, consistent with the previous report, and one for CXCR7 was also detected, confirming that these chemokine receptors are likely expressed in HEK293 cells (Fig. 5a). To confirm SDF-1α-stimulated cellular responses, HEK293 cells were treated with SDF-1α and assessed using western blotting with anti-pERK1/2 antibodies. As shown in Fig. 5b, ERK1/2 was phosphorylated by SDF-1α in HEK293 and HeLa cells, suggesting that endogenous receptors were activated by their cognate chemokine. To identify the receptor responsible for the signaling event, cell lines deprived of each of the receptors were established by using CRISPR-Cas9 technology (Additional File 1: Fig. S6a). The mRNA expression of endogenous receptors and membrane expression of HiBiT constructs of the exogenous receptors were quite similar in parent cells and the receptor knock-out cells, suggesting that CXCR4 deletion does not affect the expression of CXCR7 and vice versa (Additional File 1: Fig. S6b and S6c). SDF-1α stimulated ERK1/2 phosphorylation was still observed in CXCR7-deficient cells, but not in cells lacking CXCR4. ERK1/2 phosphorylation was not detected even in CXCR4 knockout (KO) cells transfected with the *CXCR7* gene. To examine temporal patterns of ERK1/2 phosphorylation in these cells, ligand-treated cells were harvested at different time points and assessed by western blotting. In the absence of CXCR4, ERK1/2 phosphorylation was not increased, whereas the pERK1/2 bands were strong 5 min after ligand treatment, and then decreased in both wild-type and CXCR7 KO cells. Interestingly, SDF-1α-stimulated ERK1/2 phosphorylation in CXCR7 KO cells was higher than phosphorylation in wild-type cells, suggesting that endogenous CXCR4 was activated, and the signal transduced downstream without a competitor for the ligand (Fig. 5c). The inhibitory effect of SDF-1α on β-adrenergic receptor-mediated cAMP generation was prominently reproduced in CXCR7 KO cells exogenously expressing CXCR4. In contrast, this inhibition was not observed in CXCR4 KO cells expressing CXCR7 (Fig. 5d). Overall, it is reasonable to speculate that a slight cAMP reduction in wild-type cells, regardless of CXCR7 expression, may occur by endogenous CXCR4 (Fig. 4a). Our results reinforce the hypothesis that CXCR7 was not able to activate G-proteins.

### Chemokine-stimulated β-arrestin2 recruitment at CXCR7 is mediated by GRKs

**Fig. 6:**
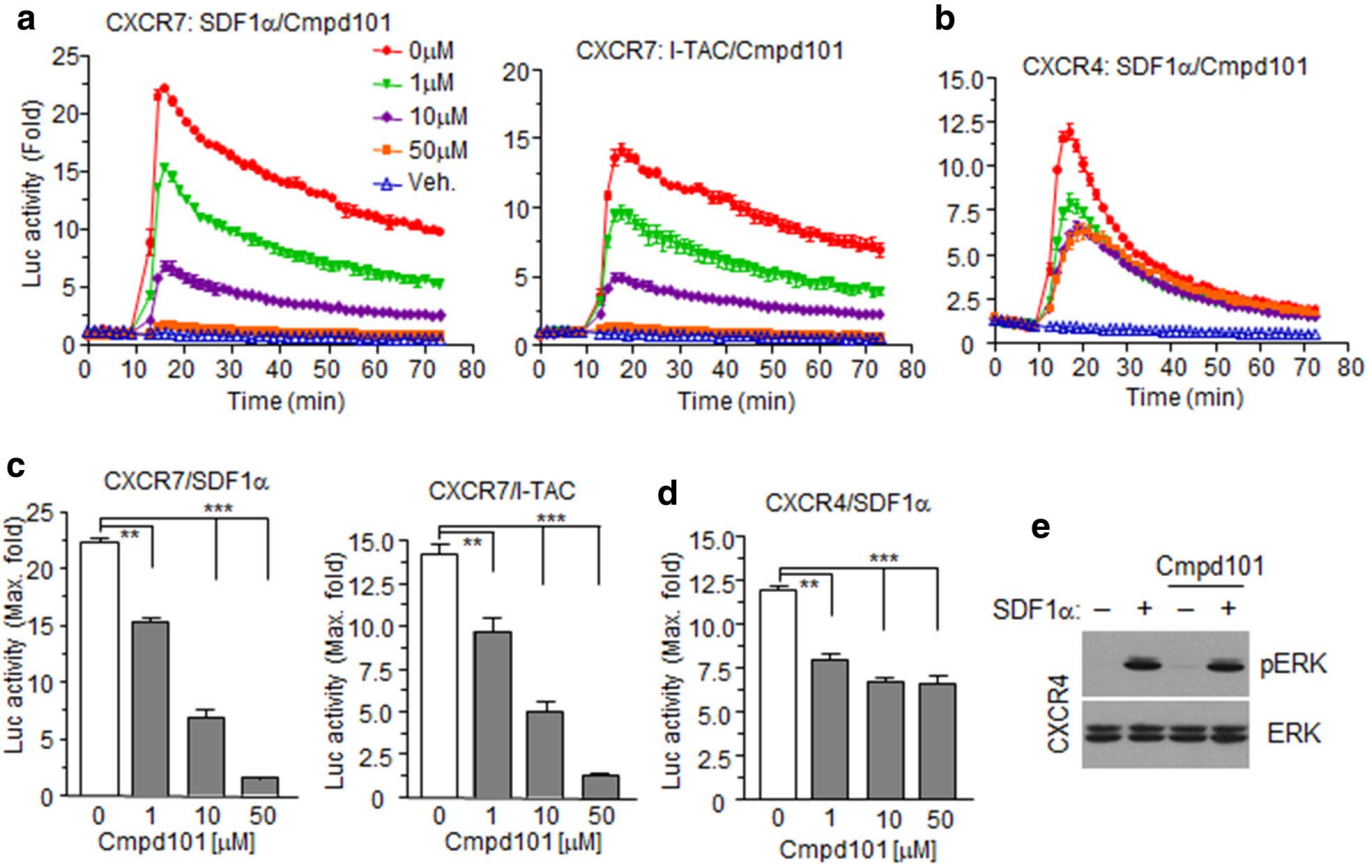
CXCR7 and CXCR4 recruitment of β-arrestin2 is GRK-dependent. **a, b** HEK293 cells expressing NanoBiT constructs for each receptor and β-arrestin2 were pre-treated with different doses of the GRK2-specific inhibitor Cmpd101, and then stimulated with their cognate chemokines. Luciferase activities were then measured. **c, d** Bar graphs show maximum luciferase activities in each group pre-treated with different doses of Cmpd101. Values are presented as the mean ± SD. **p < 0.01, **p < 0.001. **e** HEK293 cells expressing exogenous CXCR4 were pre-treated with Cmpd101, and then stimulated with SDF-1α. Cell lysates were used for western blotting with anti-pERK1/2 or ERK antibodies. Results are the average of three independent experiments

β-Arrestin recruitment to GPCRs requires phosphorylation of intracellular domains at serine or threonine residues by GPCR kinases (GRKs) [49]. As GRK2/3 and GRK5/6 subgroups are ubiquitously expressed, GRK specificity towards CXCR4 and CXCR7 was determined using a GRK2/3-selective inhibitor (Cmpd101). Signals from both chemokine-stimulated cells expressing CXCR7 and β-arrestin2 NanoBiT constructs were downregulated in a Cmpd101 dose-dependent manner, and the signals completely disappeared at a 50 μM concentration (Fig. 6a, c). In the case of CXCR4 constructs, Cmpd101 decreased luminescence signals to approximately half-maximum, even at high concentrations (Fig. 6b, d). However, SDF-1α-stimulated ERK1/2 phosphorylation in CXCR4-expressing cells was not inhibited by Cmpd101, suggesting that the β-arrestin2 contribution towards ERK1/2 phosphorylation is minimal (Fig. 6e). These results demonstrated that GRK2 and 3 were responsible for CXCR7 phosphorylation and subsequent β-arrestin2 recruitment.

### CXCR7 activates GRK2 through the β_1_ subunit of the heterotrimeric G-protein

**Fig. 7:**
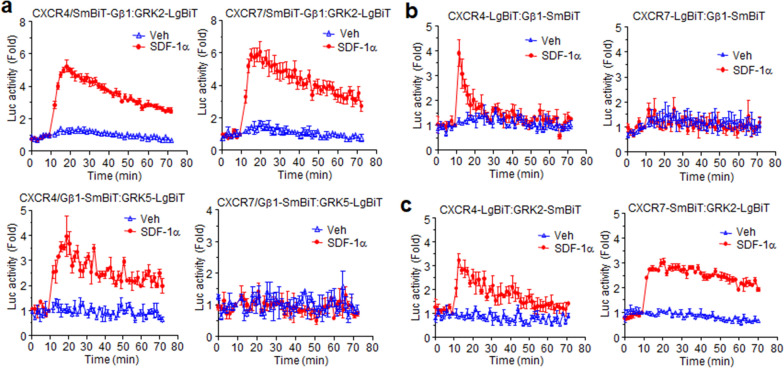
SDF-1α induces different interaction patterns between Gβ_1_ and GRK depending on the receptor. **a** Each of the chemokine receptors (CXCR4/CXCR7) was expressed in HEK293 cells together with Gβ_1_ tagged with SmBit at the N-terminal and GRK2/GRK5 tagged with LgBit at the C terminal. Luciferase activities induced by SDF-1α or not (Veh.) were then measured. **b** Cells expressing CXCR4/CXCR7 tagged with LgBiT at the C-terminal and Gβ_1_-SmBiT were treated with SDF-1α or not (Veh.), and then luminescence was measured. **c** Cells expressing CXCR4/CXCR7 tagged with LgBiT at the C-terminal and GRK2-SmBiT were treated with SDF-1α or not (Veh.), and then luminescence was measured. Results are the average of three independent experiments. Values are presented as the mean ± SD

To elucidate the molecular mechanism of how CXCR7 is able to activate GRK2 and 3 without G-protein activation, we further developed a structural complementation assay containing Gβ_1_, GRK2, and GRK5 containing the fragments LgBiT or SmBiT-tagged forms at the N- or C-terminals and chose the best combination of plasmid constructs (Additional File 1: Fig. S4). An increase in luminescence signal was observed by the interaction of SmBiT-Gβ_1_ and GRK2-LgBiT when the cells were treated with SDF-1α in the presence of CXCR4 and CXCR7 (Fig. 7a, upper graphs). SDF-1α also induced an increase in luminescence by the interaction of Gβ_1_ and GRK5 in the presence of CXCR4, but not CXCR7 (Fig. 7a, lower graphs). This was consistent with the result that the GRK2/3-specific inhibitor affected β-arrestin2 recruitment for both receptors in a dose-dependent manner (Fig. 6a, b). This molecular approach provided valuable mechanistic information about how CXCR7 can recruit β-arrestin2 via GRKs activation through the β_1_ subunit. Luminescence due to the interaction between CXCR4 with Gβ_1_ was increased by SDF-1α, but for CXCR7, its interaction with Gβ_1_ did not elicit luminescence by the chemokine, even though SDF-1α stimulation of CXCR7 induced Gβ_1_ and GRK2 interactions (Fig. 7b).

Ligand-stimulated β-arrestin2 recruitment to the chemokine receptors absolutely depends on the phosphorylation of the receptors at Ser/Thr residues in the C-terminal region and in the third intracellular loop (3ICL) [49]. According to our previous results (Fig. 6a, b), GRK2 is likely to phosphorylate both receptors. The catalytic activity of GRKs requires physical interaction with their substrates (GPCRs). The luminescence produced by each receptor with GRK2 increased depending on chemokine stimulation. Interestingly, CXCR7 registered an increased luminescence signal only in combination with GRK2-LgBiT (Fig. 7c). This observation clearly suggests that CXCR7 interacts with the heterotrimeric G-protein in a particular way that is able to generate βγ signaling, but not Gα signaling.

### Both CXCR4 and CXCR7 are necessary for SDF-1α-stimulated cell migration

**Fig. 8:**
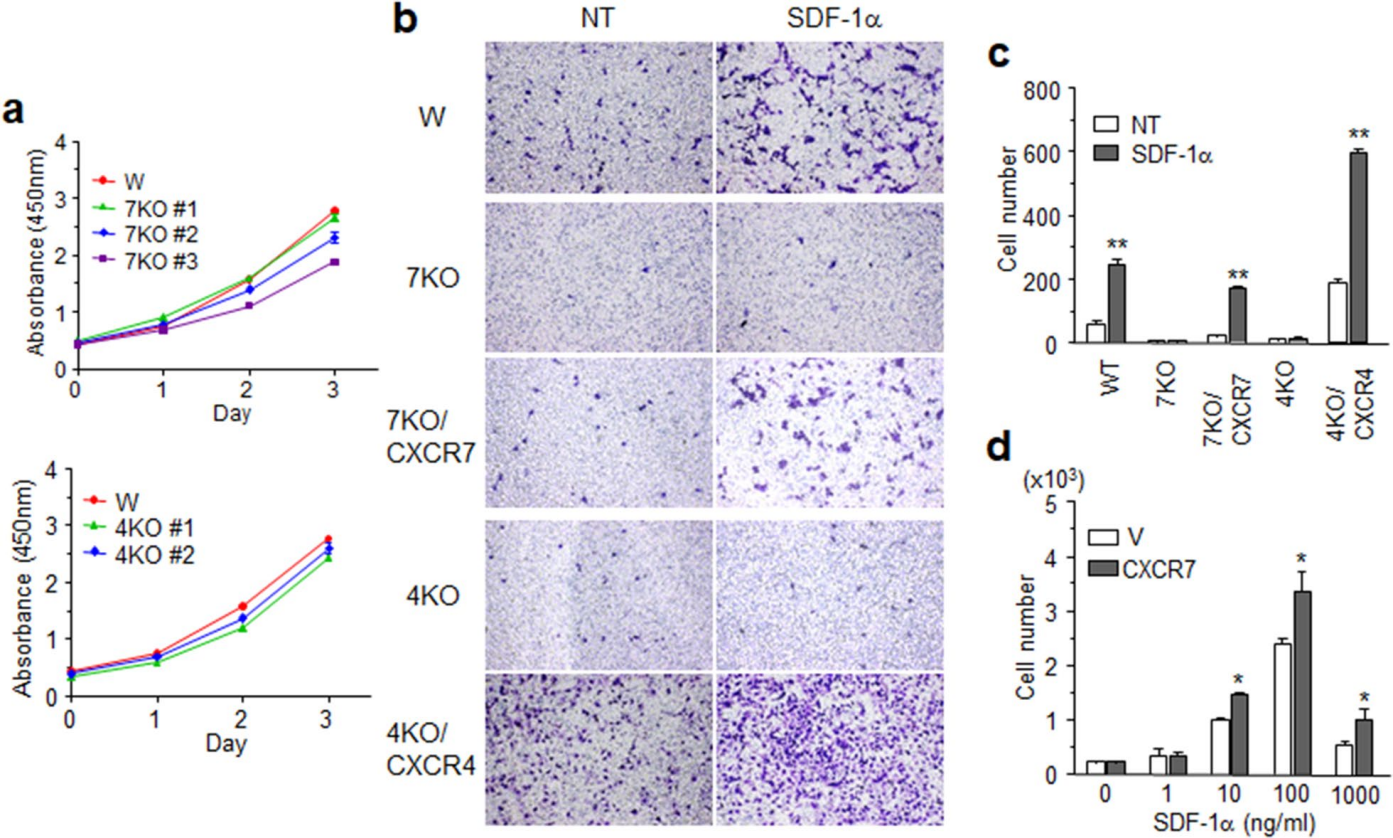
Both CXCR4 and CXCR7 are essential for cell migration. **a** Wild-type (W) and each HeLa cell clone lacking the receptors (7KO #1,2,3 and 4KO #1,2) were seeded into 96-well plates. The CCK-8 assay was performed using different plates each day. Values are means ± SDs. **b, c** Migration assay using HeLa cells. Wild-type (W) and knock-out cells infected with CXCR7 (7KO/ CXCR7) or CXCR4 (4KO/ CXCR4) were placed in the upper wells of transwell plates. 50 ng/ml of SDF-1α was added to serum-free media in the lower wells (NT: no treatment). After 24 h, migrated cells in the lower wells were stained and counted under an inverted microscope. **c** The average number of migrated cells (four different microscopic fields) is shown for the five groups. **d** Migration assay with U937 cells. CXCR7 knock-out U937 cells were re-introduced CXCR7 by infection (V: empty plasmid). Cells were placed in the upper wells, with the lower well containing increasing concentration of SDF-1α in serum-free medium. After 6 h, migrated cells into the media of the bottom wells were counted using a hemocytometer. Cell numbers are averages of migrated cells from three different wells. Results are the average of three independent experiments. Values are presented as the mean ± SD. **p < 0.01, **p < 0.001.

Regarding SDF-1α and chemotaxis, CXCR4 is known to mediate cell migration, but similar functional information about CXCR7, another receptor for SDF-1α, is not available. CXCR7 has been reported to be highly expressed in leukemic cells and to potentiate CXCR4 responses through SDF-1α in experiments using RNA interference [50], but specific effects of CXCR7 on cell migration have not been determined. As HeLa cells express both receptors and are motile toward SDF-1α, cells lacking these receptors were established as shown in Fig. 5. These receptor deficiencies did not affect cell growth in the presence of serum or SDF-1α (Fig. 8a). Migration efficiency in parental HeLa cells was high at 100 ng/ml of SDF-1α, so this same amount of SDF-1α was added to the lower wells of the migration chambers. Cells lacking either of these receptors lost the ability to migrate toward the chemokine. When CXCR7 expression was recovered in CXCR7 KO cells, migration ability was restored. Recovery of CXCR4 in CXCR4 KO cells prominently enhanced their motility, even without SDF-1α, but SDF-1α-stimulated migration was still strongly enhanced (Fig. 8b, c). This result suggests that although CXCR4 is a dominant mediator for SDF-1α-stimulated migration, CXCR7 is also essential for cell migration toward the chemokine. Chemokine receptor-mediated G-protein activation, especially Gα_i/o_ and/or Gα_12/13_, has been considered an indispensable process to endow cells with migration ability. However, CXCR7 does not mediate the activation of any Gα protein subunit, and yet the β and γ subunits in conjunction with β-arrestins somehow influence CXCR4-mediated signaling. The potentiation of cell migration by CXCR7 was also confirmed in this study; U397 cells expressing both receptors migrated toward SDF-1α in a dose-dependent manner. Moreover, increased CXCR7 expression potentiated cell migration without changing the sensitivity for the chemokine (Fig. 8d).

## Discussion

There has been a debate as to whether CXCR7 is able to activate G-proteins, or if it is only a *decoy* chemokine receptor with unknown signaling mechanisms [33]. In chemokine-stimulated cells expressing CXCR7 and GRKs subtypes, SDF-1α binding to both receptors produced interactions between Gβγ and GRKs, GRK associations and phosphorylation of receptors, and subsequent transient β-arrestin2: CXCR7 protein–protein interactions. Interestingly, even in this general process, there are distinct interactions among signaling molecules. In this study, signaling properties of the two SDF1a receptors were deeply investigated and compared each other by with structural complementation assays combined with different expression levels controlled by various promoters as well as classical pathway analysis tools such as ERK phosphorylation, calcium assay, and cAMP generation. Both receptors mediated interactions between Gβ_1_ and GRK2, while GRK5 interaction with Gβ_1_ was detected in the presence of CXCR4, but not CXCR7. β-arrestin2 recruitment in the presence of GRK2/3 inhibitor confirmed the strict control of GRKs in receptor-β-arrestin interactions. Given these results, the SDF-1α-CXCR7 complex dissociates Gβγ subunits from Gα_i_ in such a manner that Gβγ selectively activates GRK2, but not GRK5, even though it may not stimulate GDP release from Gα_i_.

NanoBiT assay may be a good tool to determine the molecular interaction properties since all proteins could be constructed as N-ter or C-ter tagged forms of the Nluc fragments and applied to the assay in different combinations. Regarding interactions between Gβ_1_ and GRK subfamilies, SmBiT-Gβ_1_ interacts with GRK2-LgBit, whereas Gβ_1_-SmBiT binds to GRK5-LgBiT upon CXCR4 activation as shown in Fig. 7a, suggesting that GRK2 and GRK5 bind to different regions of Gβ_1_. Unlike CXCR4, SDF-1α-bound CXCR7 may expose GRK2 binding regions of Gβ_1_ but block the interaction between Gβ_1_ and GRK5 possibly by still occupying GRK5 binding site in Gβ_1_, leading to the specificity for GRK2. While this structural complementation technology offers some interesting insights into molecular interaction, it may also have some limitations to read out all interactions in the molecular networks, since not all molecular interactions facilitate close access between the fragments. In the present study, the interaction between CXCR7 and Gβ_1_ was not confirmed using this assay, as the luciferase activities were not increased in any combination of the NanoBiT constructs. This drawback would be relieved by incorporation of the Nluc fragments inside of the protein without destroying the functional and structural integrity of the molecules.

Previous research has suggested that CXCR4/CXCR7 cross-talk occurs through heterocomplex formation [35, 42, 51]. However, we could not find any evidence of the direct interactions of them in our cross-linking experiments and novel structural complementation assay. Overexpression of proteins, driven by strong promoters, often leads to non-specific interactions between molecules that do not reflect physiological protein functions. Therefore, any conflict between the present results and previous reports may be ascribed to artificial expression levels that do not reflect the physiological environment in which the receptors function. Besides, our results indicate that they may be expressed in the plasma membrane as homodimers for effective stimulation of downstream signaling. Although it is hard to determine how many receptor molecules are expressed as monomer or homodimer, the functionality of homodimers was confirmed in the competition assay. SDF-1α-stimulated association of CXCR7-LgBiT and SmBiT-β-arrestin2 was enhanced in the presence of a small number of intact CXCR7 driven by HSV-TK promoter, indicating that absolute dimer number of CXCR7-LgBiT (as CXCR7-LgBiT/CXCR7-LgBiT and CXCR7/CXCR7-LgBiT to recruit SmBiT-β-arrestin2 was increased. However, the enhancement of the NanoBiT activities was not observed in the combination of CXCR7-LgBiT and intact CXCR4 or CXCR4-LgBiT and intact CXCR7. This may be explained by simple competition to SDF-1α without heterodimerization.

According to the primer-specific RT-PCR results, both CXCR4 and CXCR7 may be expressed in most established cells, which makes understanding the functional roles of each receptor difficult. CRISPR–Cas9-based gene deletion is a useful tool to reveal the function of the gene products. Cells lacking each receptor were developed by this technology and used for cell-response analyses to SDF-1α stimulation. Chemokine-dependent ERK1/2 phosphorylation did not occur only in CXCR4-deficient cells, indicating that CXCR4-mediated Gα_i_ activation was essential for ERK1/2 phosphorylation. In many reports, β-arrestin2 is considered to be another mediator of GPCR-stimulated ERK1/2 phosphorylation [52–54]]. However, this was not reproduced in our chemokine-stimulated cellular responses because even CXCR7 overexpression in CXCR4 KO cells had no effect on ERK1/2 phosphorylation.

SDF-1α is a well-known chemokine that induces mobilization of various cells and is involved in inflammation, angiogenesis, and cancer metastasis [55–57]. This chemokine stimulated HeLa cell migration, but no chemotaxis was observed in cells lacking each of the receptors. However, chemotaxis was restored by reconstituted receptor expression. This result supports the idea that CXCR7 is not a simple chemokine scavenger, but instead an essential mediator for SDF-1α-stimulated migration, even though CXCR4 could be predominant. The role of CXCR7 in cell migration is not likely due to the rapid elimination of SDF-1α by high-affinity binding because the receptor had no effect on cell sensitivity to the chemokine. Given that CXCR7 potentiated cell migration toward SDF-1α, CXCR7 may be involved in inflammation and metastasis together with CXCR4, although the precise mechanism for this remains to be elucidated.

In the present study, we defined distinct signaling pathways mediated by CXCR4 and CXCR7 by using a series of novel structural complementation assays as well as CRISPR-Cas9. As a biased receptor, CXCR7 recruited β-arrestin2 in response to SDF-1α stimulation, which was mediated by Gβ_1_ and GRK2, without Gα_i_ activation. The signaling by CXCR4 and CXCR7 potentiated cell motility toward SDF-1α, supporting the idea that both receptors are potential therapeutic targets for pathological conditions, such as inflammation and cancer.

## Materials and methods

### Materials

All chemokines were obtained from PeproTech (Rocky Hill, NJ, USA). The NanoBiT starter kit, containing the plasmids and all reagents for the protein interaction assay, was from Promega (Madison, WI, USA). The pBiT3.1 plasmid, pGlo22F plasmid, and all reagents for these plasmid-related assays were also purchased from Promega. The pcDNA3.1 expression vector was purchased from Invitrogen (San Diego, CA, USA). The SRE-Luc vector which contains four copies of the serum response element (SRE; CCATATTAGG) was acquired from Stratagene (La Jolla, CA, USA). Anti-HA antibodies and agarose beads conjugated with anti-FLAG antibodies were from Sigma-Aldrich (St. Louis, Mo, USA). Anti-ERK (cat. no. 4695) and anti-pERK (Thr202/Tyr204) (cat. no. 4370) antibodies were from Cell Signaling Technology (Beverly, MA, USA). Anti-GFP (cat. no. sc-8432), anti-β-actin (cat. no. sc-9996), and all secondary antibodies were from Santa Cruz Biotechnology (Santa Cruz, CA, USA). All primers for gene cloning and PCR and related materials were obtained from Cosmo Genetech Co., Ltd. (Seoul, Korea), and the DNA sequencing was conducted by Macrogen (Seoul, Korea). Unless otherwise stated, all reagents were purchased from Sigma-Aldrich.

### Cell culture

HEK293 and HeLa cells were obtained from the American Type Culture Collection (ATCC, Manassas, VA, USA). All cell lines were maintained in Dulbecco’s modified Eagle’s medium (DMEM) supplemented with 10% fetal bovine serum (FBS), 100 IU/ml penicillin G, and 100 μg/ml streptomycin (Invitrogen, Carlsbad, CA, USA). Gα_12/13_-knockout HEK293 cells were a kind gift from Professor Asuka Inoue, Tohoku University.

### Plasmid construction

The CMV promoter sequence in pcDNA3.1 vector was substituted with promoters from the *Ubiquitin C* gene (UbiC) or *HSV-TK* gene (HSV-TK) to develop a regulated expression system. *CXCR4*, *CXCR7*, and *GFP* genes were inserted into these vectors. The fragments of *Nano-Luciferase* gene in NanoBiT vectors from the company were inserted into a multicloning site in UbiC. All genes for the NanoBiT assay were constructed as N-terminal or C-terminal tagged forms in the vector containing the UbiC promoter. The chimeric receptor genes were generated by overlap PCR using primers and inserted into the NanoBiT vector. Either the *CXCR4* or *CXCR7* gene was inserted into the FG12 vector to generate lentivirus.

### RT-PCR

Total RNA was extracted from cells using TRIzol (Invitrogen) according to the manufacturer’s instructions. Next, 3 μg of RNA was reverse transcribed using M-MLV Reverse transcriptase to generate cDNA (Promega, Madison, WI, USA). The PCR reaction mixture contained cDNA in the presence of Taq DNA polymerase, buffer, dNTPs, and primer pairs. The primer pairs for the housekeeping gene beta-actin (Forward: 5′-AGAAAATCTGGCACCACACC-3′; Reverse: 5′-CCATCTCTTGCTCGAAGTCC-3′) generated PCR products of 435 bp. The human CXCR7 primers (Forward: 5′-CAGCAGAGCTCACAGTTGTTG-3′; Reverse: 5′-GAGCAGGACGCTTTTGTTGG-3′) and human CXCR4 primers (Forward: 5′-GGCCAGCCAGCACCTATTTG-3′; Reverse: 5′-TGGCTTTGCCCCCTTGAAA-3′) amplified fragments of 269 bp and 254 bp, respectively. The PCR reaction was performed on a thermal cycler (SimpliAmp Thermal Cycler, Thermo Fisher Scientific) using the following conditions: 95 °C, 5 min; 30 cycles (95 °C, 30 s; 58 °C, 30 s; 72 °C, 40 s); 72 °C, 7 min. PCR products were separated on a 2% agarose gel by electrophoresis and imaged.

### Structural complementation assay based on NanoBiT technology

HEK293 cells were seeded into 96-well microplates at the density of 2.0 × 10^4^ cells/ well. The next day, 50 ng of receptor plasmid (receptor fused with LgBiT or SmBiT) and 50 ng of β-arrestin2 plasmid (attached with SmBiT or LgBiT in N-terminal or C-terminal) were mixed with 0.2 μl Lipofectamine 2000 (Invitrogen, Carlsbad, CA) and added to the cells. The other steps of transfection were carried out following the manufacturer’s instructions. This formulation was used for all the other two-gene combinations. For three-gene combinations, 30 ng of each gene preparation was used for transfections. 24 h later, before measuring luminescence, cells were stabilized for 10 min at room temperature by replacing the media with 100 μl of Opti-MEM. Then, 25 μl Nano-Glo Live Cell Reagent (furimazine) was added to each well, and baseline luminescence was measured for the first 10 min. Finally, cells were stimulated using 10 μl of SDF-1α or I-TAC at a concentration of 100 ng/ ml, and cell-plate measurements were continued for 1 h. These procedures were conducted using a luminometer (BioTek Instruments, Inc., Winooski, VT, USA).

### HiBiT assay

The expression of receptors on cell membranes was detected using the Nano-Glo HiBiT extracellular system (Promega, Madison, USA). HEK293 cells were seeded into 96-well plates at a density of 2.0 × 10^4^ cells per well. The next day, cells were transfected with a mixture of DNA constructs of SmBiT-receptors: pBiT3.1 N (0.5 ng, 5 ng, or 50 ng) and 0.2 μl of Lipofectamine 2000. After 24 h, 100 μl of Nano-Glo HiBiT extracellular reagent (1 μl of LgBiT protein + 2 μl substrate + 97 μl of Nano-Glo HiBiT buffer) was added to each well. The assay plate was then left to equilibrate for 4 min at room temperature without mixing. Luminescence values were measured using the Synergy 2 Multi-Mode Microplate Reader (BioTek, Winooski, VT, USA).

### Cellular imaging

HEK293 cells were seeded onto poly-L-lysine-coated cover glasses. The next day, cells were transfected with plasmids containing receptors in different forms or β-arrestin2-GFP by Lipofectamine 2000. SDF-1α was applied to the cells for 30 min after overnight starvation. After washing with PBS, cells were fixed, and GFP signals were observed using a LSM800 confocal microscope (Carl Zeiss Microimaging Inc., Zena, Germany). For pulse labeling of surface receptors described previously [41], cells expressing HA-tagged receptors were kept at 4 °C for 60 min after the medium was replaced by ice-cold Opti-MEM (Invitrogen) containing anti-HA antibodies. Cells were washed with PBS and incubated with Opti-MEM containing SDF-1α at 37 °C for 30 min to allow receptor internalization. Then, cells were fixed, permeabilized, and HA signals were detected using FITC-conjugated goat anti-mouse IgG. Images were obtained using the confocal microscope.

### Immunoprecipitation and western blot analyses

Cells were lysed by adding lysis buffer (150 mM NaCl, 50 mM Tris–HCl pH 7.5, 10 mM KCl, 1% Triton X-100, 10 mM NaF, 5 mM Na_3_VO_4_) complemented with protease inhibitor cocktail (Roche, Indianapolis, IN, USA). Protein quantification was performed using a Bradford protein assay kit (Bio-Rad, Hercules, CA, USA). Cell lysates were then denatured with SDS sample buffer and an equal amount of protein was separated on 10% polyacrylamide gels and then transferred to nitrocellulose membranes for immunoblotting. The membranes were blocked with 5% nonfat dry milk in Tris-buffered saline with Tween 20 (TBST) and probed with appropriate antibodies. Finally, the signal by HRP-conjugated secondary antibodies was developed using an enhanced chemiluminescence (ECL) kit (GE Healthcare Life Sciences, Marlborough, MA, USA).

For immunoprecipitations, HEK293 cells were transfected with HA-tagged CXCR4 or CXCR7. 36 h after transfection, cells were washed with cold PBS and lysed with lysis buffer containing protease inhibitor cocktail (Roche). For the cross-linking experiments, cells were washed with PBS twice and incubated with PBS containing 2 mM Bissulfosuccinimidyl substrate (BS^3^) for 30 min. The reaction was stopped by adding Tris-buffered saline, and cells were then washed again with the same buffer and lysed with lysis buffer. The lysates were centrifuged, and the supernatants were incubated with anti-FLAG antibody-conjugated beads. The beads were washed at least four times with the lysis buffer and the bound proteins in SDS sample buffer were subjected to SDS-PAGE, followed by immunoblotting with the appropriate antibodies.

### cAMP assay

HEK293 cells were seeded into 96-well microplates at a density of 2.0 × 10^4^ cells/well. The next day, 60 ng of pcDNA3.1/ CXCR7 or pcDNA3.1/ CXCR4 and 40 ng of pGlo22F plasmid were added to each well. The plate was incubated in the 5% CO_2_ incubator at 37 °C to let proteins express for 36 h. Before performing the assay, cells were incubated in an equilibration medium containing 10% v/v dilution of the Glosensor cAMP reagent stock solution using CO_2–_independent medium. After 1 h of incubation, cells were stabilized for 10 min at RT. Cells were treated with vehicle or 100 ng/ml SDF-1α. After 10 min, isoproterenol was added at 10 μM following by luminescence measurement for 50 min. Luminescence values were measured using the Synergy 2 Multi-Mode Microplate Reader (BioTek, Winooski, VT, USA).

### Detection of intracellular calcium increase

We developed a new method to measure intracellular calcium change using NanoBiT technology [58]. HEK293 cells stably expressing Gα_qi_ chimera were seeded in 96-well plates at a cell density of 2×10^4^ cells/well. The next day, 30 ng of receptor plasmids, 30 ng of plasmids containing calmodulin tagged with SmBiT at C-terminal, and 30 ng of plasmids containing MYLK2S fused to LgBiT at the N-terminal were mixed with 0.2 μl Lipofectamine 2000 (Invitrogen, Carlsbad, CA) and added to the plated cells. Following transfection, steps were performed according to the manufacturer’s instructions. After 24 h, media was replaced with 100 μl of Opti-MEM, and cells were stabilized for 10 min at room temperature before measuring the luminescence. Then, 25 μl of Nano-Glo Live Cell Reagent (furimazine) was added to each well, and basal luminescence was measured using a luminometer (BioTek Inc., Winooski, VT, USA) for the first 10 min. Finally, cells were stimulated by adding 10 μl of SDF-1α at a final concentration of 100 ng/ ml to each well, and the real-time change of luminescence was measured for 30 min.

### Reporter gene assay

HEK293 cells exogenously expressing Gα_qi_ construct were seeded into 48-well plates at a density of 2.5 × 10^4^ cells/well. The next day, a mixture of 75 ng of pcDNA3.1/CXCR7 (or pcDNA3.1/CXCR4, pcDNA3.1/CXCR3), 75 ng of SRE-Luc reporter gene plasmids, and 0.3 μl of Lipofectamine 2000 was added per well, according to the manufacturer’s instructions. The next day, cells were maintained in serum-free DMEM overnight. After approximately 36 h since transfection, the cells were treated with SDF-1α (100 ng/ml), I-TAC (100 ng/ml), or VUF11207 (100 nM) for 6 h. Cells were then lysed with 100 μl of lysis buffer, and the luciferase activity of cell extracts was measured using a luciferase assay system, following the standard protocol for the Synergy 2 Multi-Mode Microplate Reader (BioTek Inc., Winooski, VT, USA).

### Establishment of knockout cells by CRISPR-Cas9

To establish cells lacking receptors, four potential target sequences for each gene were chosen using a guide design program from the Zhang Lab (https://www.zlab.bio/guide-design-resources). The two-strand oligos were annealed and inserted into the pRG2 vector, and 49 nucleotides including the target site were inserted into the pMRS surrogate vector. These two vectors were introduced into HEK293 cells with p3S-Cas9 plasmids and examined guide efficiency by genomic DNA PCR with appropriate primers and T7E1 treatment. The efficient guide vectors (CXCR4: TACACCGAGGAAATGGGCTCAGG, CXCR7: GGAACTTCTCGGACATCAGCTGG), surrogate vector, and p3S-Cas9 were transfected into the cells. Potential gene knockout cells were isolated by MACSelect Kk MicroBeads (Miltenyi Biotec, Bergisch Gladbach, Germany) and transferred to 96-well plates at 0.5 cells/ well. Gene deletion was confirmed by genomic DNA PCR and T7E1 analysis. PCR products were inserted into pGEM-T easy vector. After transformation, the isolated DNAs from 10 clones of *E. coli* were sequenced to confirm gene modification.

### Growth assay

HeLa cells (2000 cells/well) lacking each of the receptors were seeded into 4 different 96-well plates containing complete media for 24 h. After every 24 h, cells in each plate were incubated with 10 μl of CCK-8 solution for 2 h, and the absorbance of each well was then measured at 450 nm using a microplate reader. Cell growth was measured using the Cell Counting Kit-8 (CCK-8) from Dojindo Molecular Technologies, Inc. (Rockville, MD, USA) following the manufacturer's instructions.

### Migration assay

HeLa CXCR4 KO and CXCR7 KO cells were first infected with FG12/CXCR4 or FG12/CXCR7 virus supernatants using 5 μg/ml polybrene. Chemotaxis assays were performed by using transwell plates with 8-μm pore size (Corning Inc. Corning, NY, USA). The inserts were filled with 2.5 × 10^4^ cells in 100 μl serum-free DMEM. For the lower wells, 650 μl of serum-free DMEM with SDF-1α (50 ng/ml) was added. The cells were kept in a 37 °C incubator containing 5% CO_2_. After 24 h, non-migrated cells in the upper chamber were removed by a wet cotton swab. Cells that migrated were fixed with 4% paraformaldehyde, stained using hematoxylin and eosin, and then counted in four high-power microscope fields (100 ×).

U937 cells infected with FG12/CXCR7 virus were applied to the chemotaxis assay. The upper wells of transwell plates (8-μm pore size) were filled with 2.5 × 10^4^ cells in 100 μl serum-free RPMI, and the lower wells were filled with 650 μl of serum-free RPMI containing different concentrations of SDF-1α. After 6 h, cells that had migrated to the lower wells were counted using a hemocytometer.

### Statistical analysis

Unpaired Student’s *t* tests or ANOVA using PRISM5 software (GraphPad; La Jolla, CA, USA) were used for statistical analyses. Group means were further analyzed using Bonferroni’s multiple-comparison tests. The data were presented as the mean ± SD, and all experiments were performed as triplicates unless otherwise indicated.

## Supplementary information

**Additional File 1: Fig. S1.** Ligand-stimulated real-time luciferase activities in HEK293 cells expressing different combinations of NanoBit constructs ofCXCR7 and β-arrestin2. ** Fig. S2.** Ligand-stimulated real-time luciferase activities in HEK293 cells expressing different combinations of NanoBit constructs of β-arrestin2 with CXCR4 or CXCR3. ** Fig. S3.** Receptor internalization assay using NanoBit constructs. (a) Cells expressing receptor-LgBiT and SmBiT-FYVE domain of EEA1 were treated with SDF-1α and the luciferase activities were measured in real-time. (b) Cells expressing receptor-SmBiT and LgBiT-CAAX sequence were used in the NanoBit assay. ** Fig. S4.** Optimization of NanoBit construct combinations of Gb1 and GRKs. a and b showed luciferase activities in cells expressing different combinations of NanoBiT constructs. ** Fig. S5.** Optimization of NanoBit construct combinations of receptor and GRK2. ** Fig. S6.** Generation of cells lacking receptors using CRISPR system. (a) Genomic DNA PCR products from cells established with CRISPRCas9were cloned and sequenced. Red color designates guide RNA target regions. (b) RT-PCR products of either CXCR4 or CXCR7 were compared in wild-type and receptor KO HeLa cell clones. β-actin products were used as the control. (c) Membrane expression of exogenous receptors was not affected by deletion of CXCR4 or CXCR7. HiBiT constructs of the receptors were expressed in wild-type and receptor KO of HEK293 and HeLa cells, and the cells were applied to the HiBiT assay.

## Data Availability

Please contact the corresponding author for data on reasonable request.

## Abbreviations

CXCR: C-X-C chemokine receptor
ACKR: Atypical chemokine receptor
SDF-1α: Stromal cell-derived factor 1α
I-TAC: Interferon-inducible T-cell alpha chemoattractant
NanoBiT: NanoLuc Binary Technology
GFP: Green Fluorescent Protein
SRE: Serum response element
GRK: G-protein coupled receptor kinase
