# Supplementary material for: CXCR7: a β-arrestin-biased receptor that potentiates cell migration and recruits β-arrestin2 exclusively through Gβγ subunits and GRK2
Source: Cell Biosci. 2020 Nov 23;10:134. doi: 10.1186/s13578-020-00497-x (PMC7686738; doi:10.1186/s13578-020-00497-x)
Supplement: Supplementary file 1 — Additional File 1: Fig. S1. Ligand-stimulated real-time luciferase activities in HEK293 cells expressing different combinations of NanoBit constructs ofCXCR7 and β-arrestin2. Fig. S2. Ligand-stimulated real-time luciferase activities in HEK293 cells expressing different combinations of NanoBit constructs of β-arrestin2 with CXCR4 or CXCR3. Fig. S3. Receptor internalization assay using NanoBit constructs. (a) Cells expressing receptor-LgBiT and SmBiT-FYVE domain of EEA1 were treated with SDF-1α and the luciferase activities were measured in real-time. (b) Cells expressing receptor-SmBiT and LgBiT-CAAX sequence were used in the NanoBit assay. Fig. S4. Optimization of NanoBit construct combinations of Gb1 and GRKs. a and b showed luciferase activities in cells expressing different combinations of NanoBiT constructs. Fig. S5. Optimization of NanoBit construct combinations of receptor and GRK2. Fig. S6. Generation of cells lacking receptors using CRISPR system. (a) Genomic DNA PCR products from cells established with CRISPRCas9were cloned and sequenced. Red color designates guide RNA target regions. (b) RT-PCR products of either CXCR4 or CXCR7 were compared in wild-type and receptor KO HeLa cell clones. β-actin products were used as the control. (c) Membrane expression of exogenous receptors was not affected by deletion of CXCR4 or CXCR7. HiBiT constructs of the receptors were expressed in wild-type and receptor KO of HEK293 and HeLa cells, and the cells were applied to the HiBiT assay. [file 13578_2020_497_MOESM1_ESM.pdf]

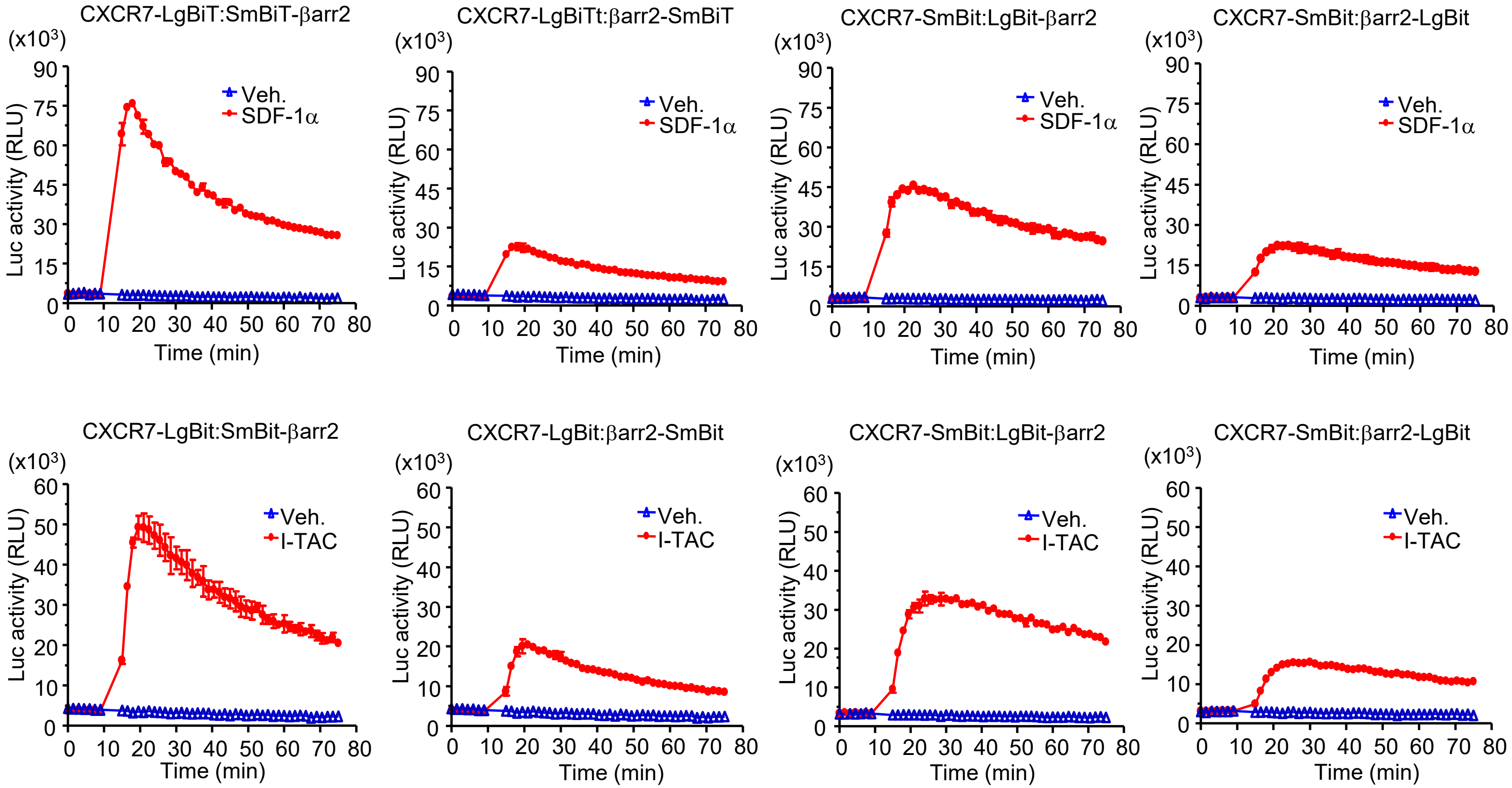

Fig. S1 Ligand-stimulated real-time luciferase activities in HEK293 cells expressing different combination of NanoBit constructs of CXCR7 and β-arrestin2

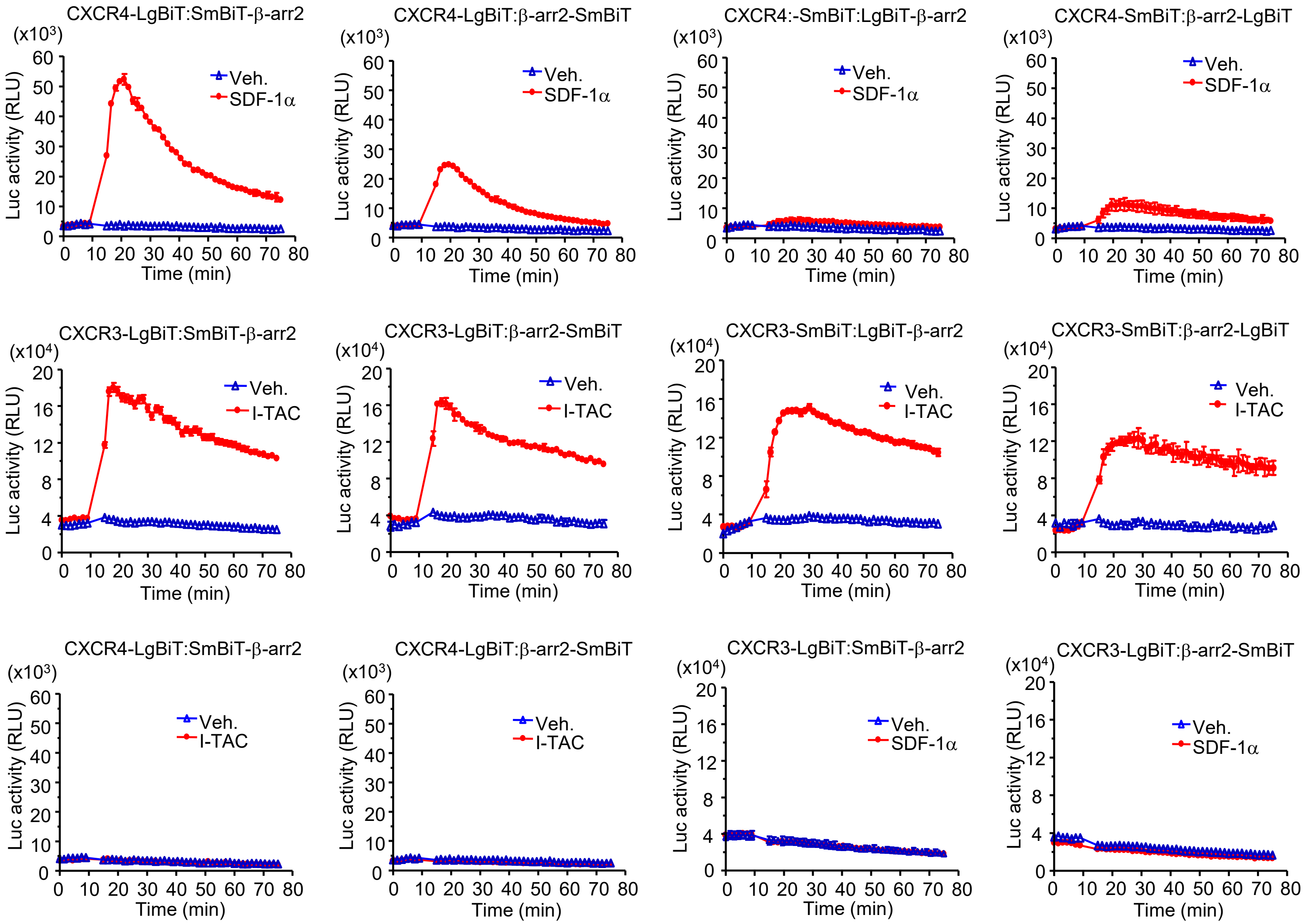

Fig S2 Ligand-stimulated real-time luciferase activities in HEK293 cells expressing different combination of NanoBit constructs of β-arrestin2 with CXCR4 or CXCR3

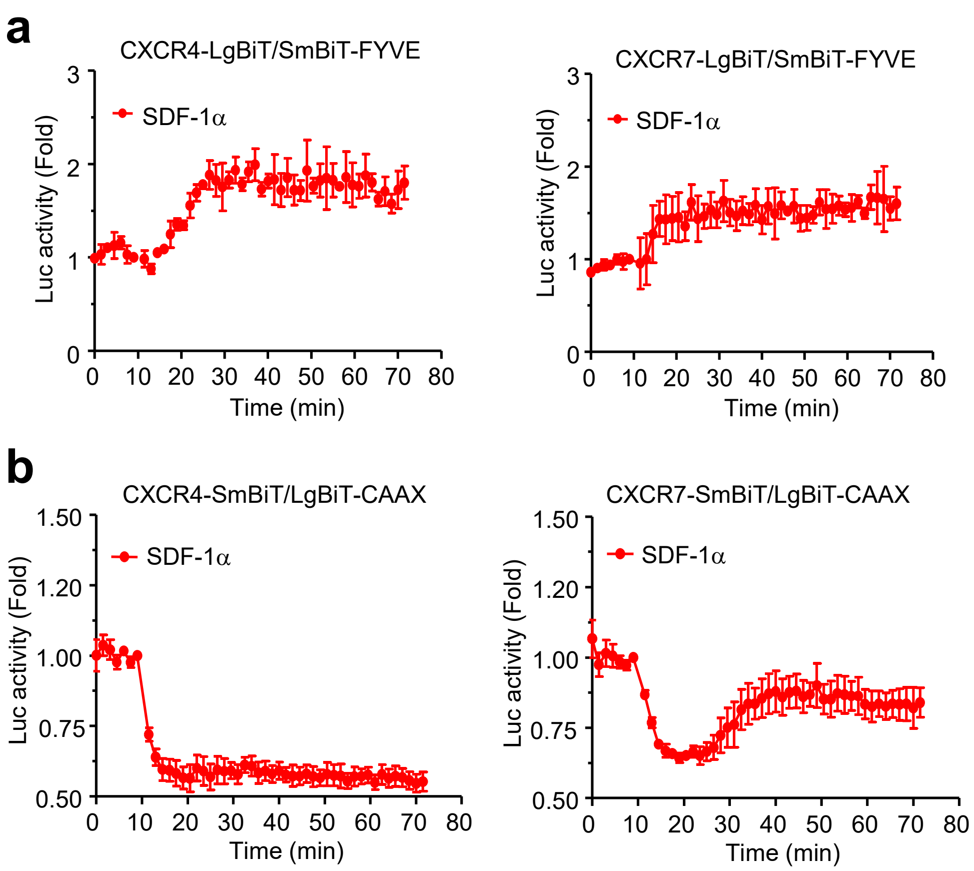

Fig S3 Receptor internalization assay using NanoBit constructs. (a) Cells expressing receptor-LgBiT and SmBiT-FYVE domain of EEA1 were treated with SDF-1α and the luciferase activities were measured in real time. (b) Cells expressing receptor-SmBiT and LgBiT-CAAX sequence were used the NanoBit assay.

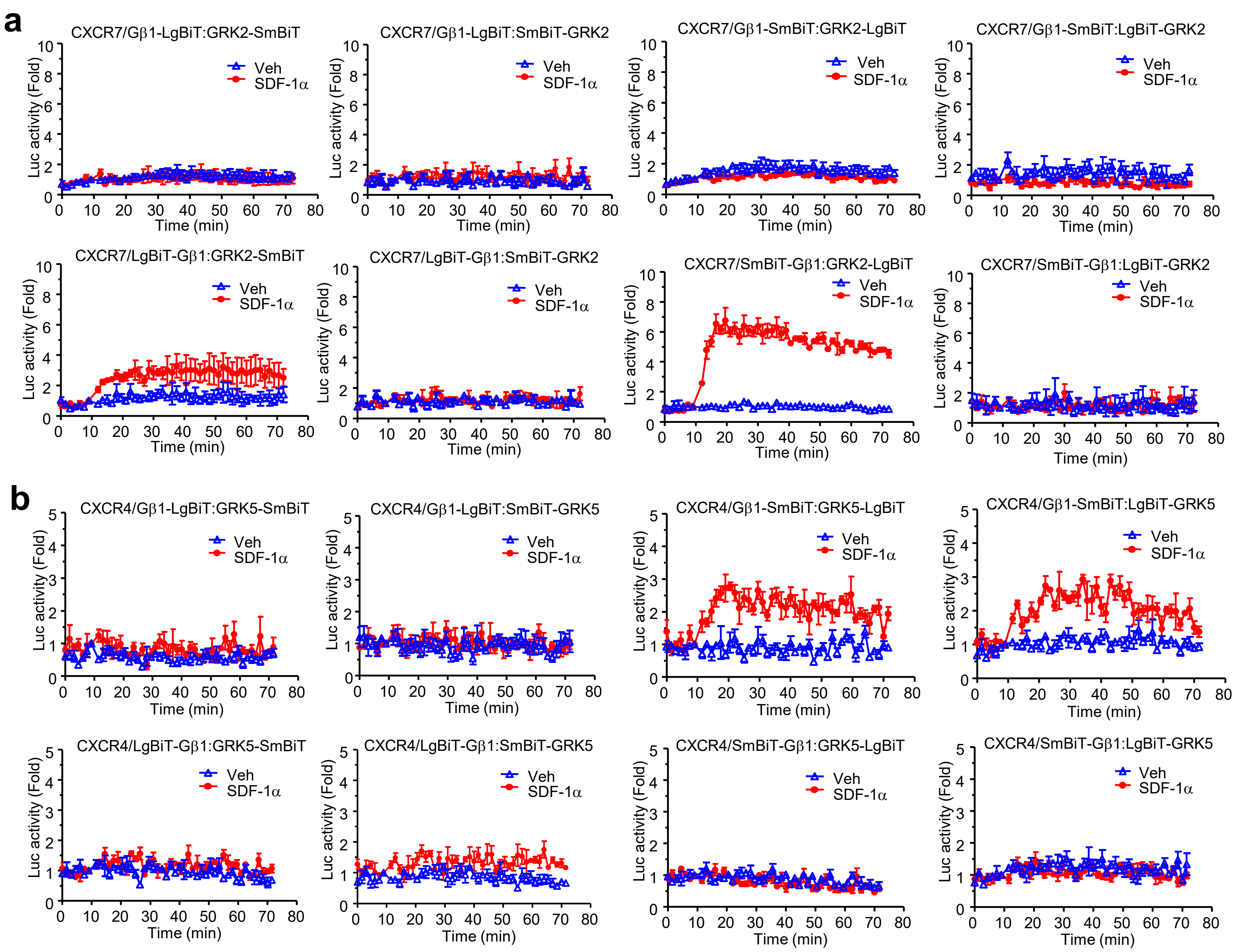

Fig S4 Optimization of NanoBit construct combinations of Gβ1 and GRKs. a and b showed luciferase activities in cells expressing different combinations of NanoBiT constructs

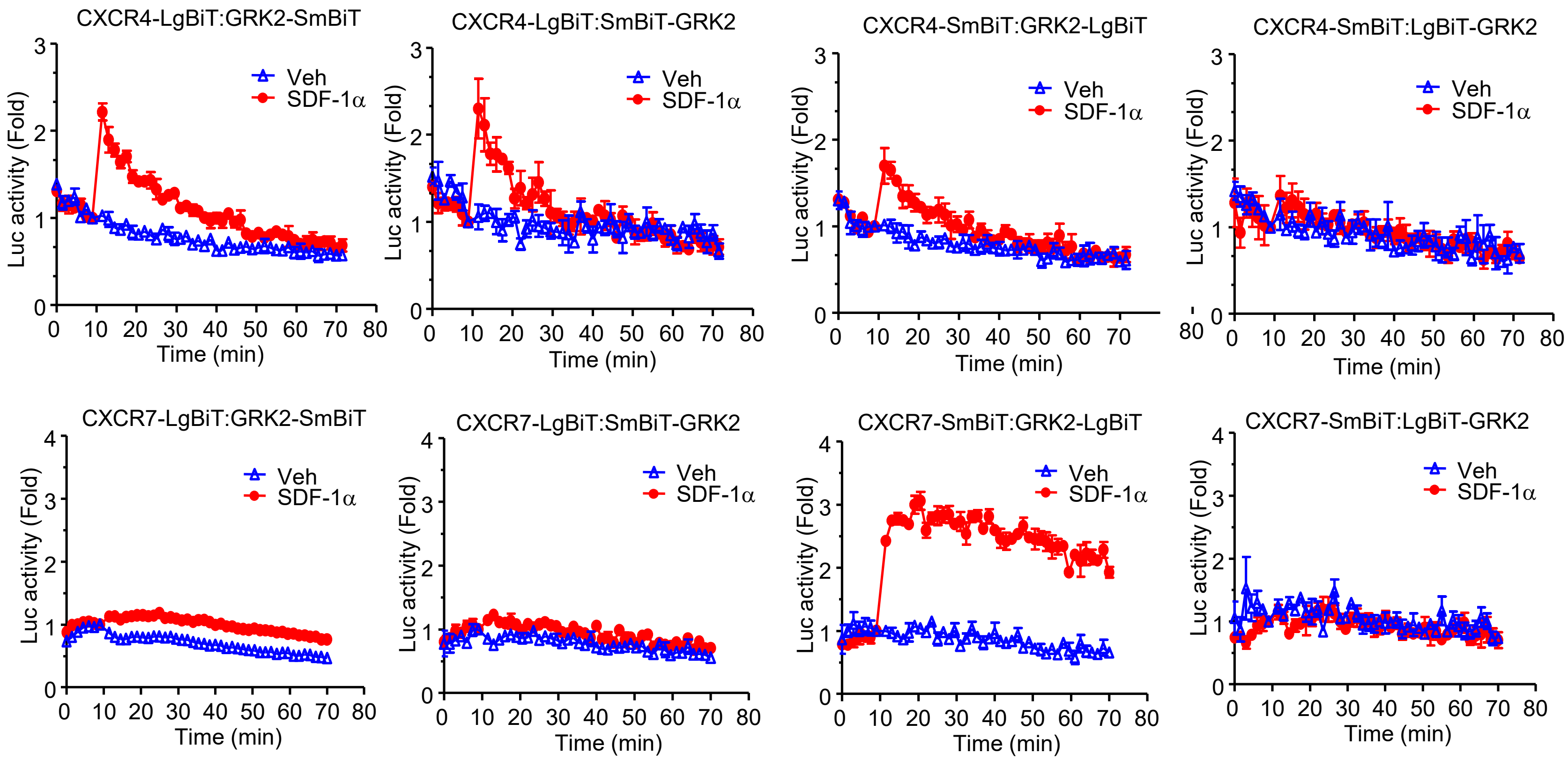

Fig S5 Optimization of NanoBit construct combinations of receptor and GRK2.

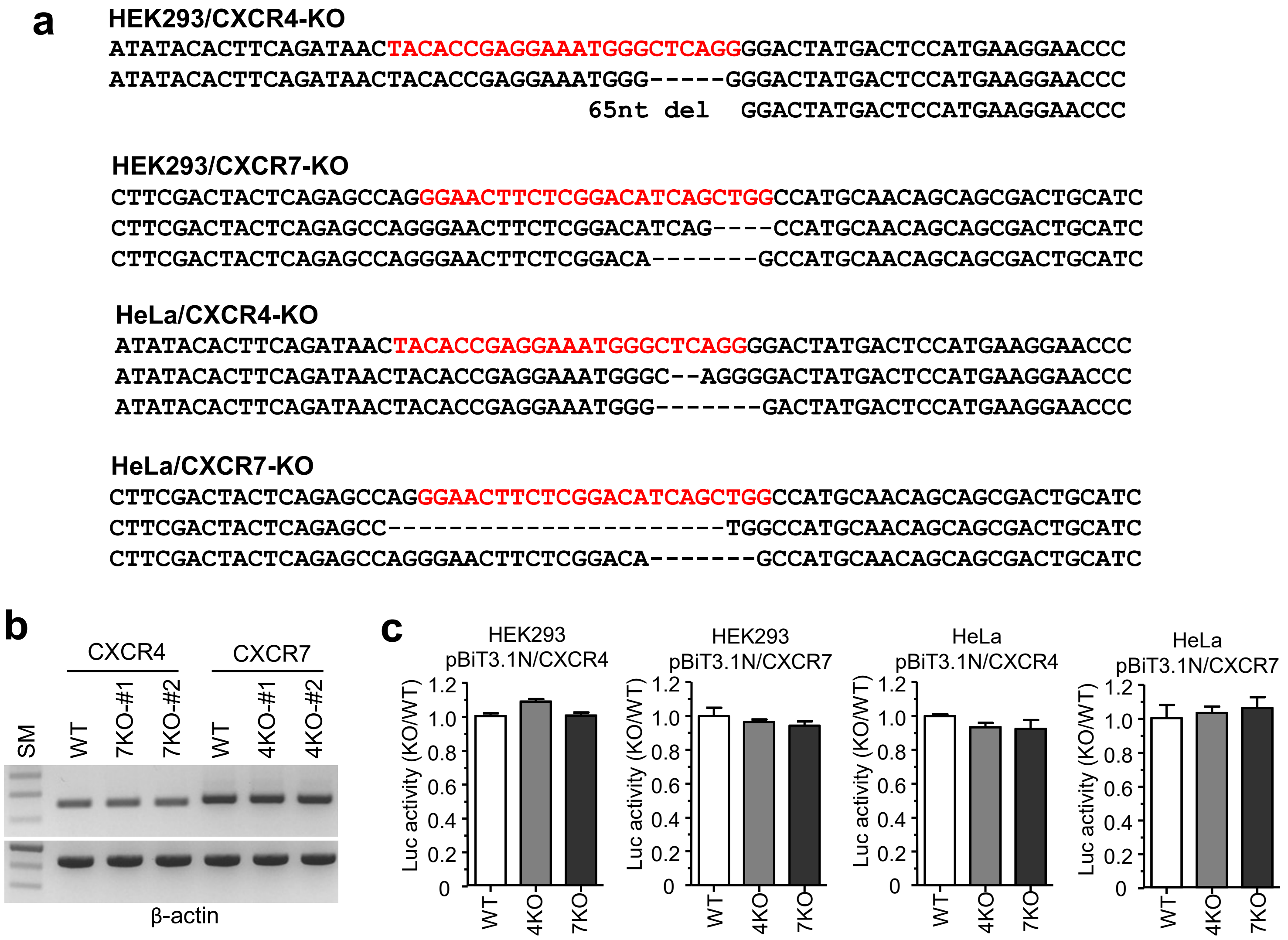

Fig S6 Generation of cells lacking receptors using CRISPR system. (a) Genomic DNA PCR products from cells established with CRISPR-Cas9 were cloned and sequenced. Red colors designates guide RNA target regions. (b) RT-PCR products of either CXCR4 or CXCR7 were compared in wild type and receptor KO HeLa cell clones. β-actin products were used as a control. (c) Membrane expression of exogenous receptors were not affected by deletion of CXCR4 or CXCR7. HiBiT constructs of the receptors were expressed in wild type and receptor KO of HEK293 and HeLa cells, and the cells were applied to HiBiT assay.
